# Microbiota–immune dysregulation in cervical cancer patients from Western Mexico: linking gut dysbiosis and NK cell exhaustion as promising biomarkers

**DOI:** 10.3389/fimmu.2025.1637098

**Published:** 2025-10-31

**Authors:** Ksenia Klimov-Kravtchenko, Tonatiuh Abimael Baltazar-Díaz, Jesse Haramati, Paula Alejandra Castaño-Jiménez, Fabiola Solorzano-Ibarra, Jose Manuel Rojas-Diaz, Nadia Tatiana Garcia-Barrientos, Jose Alfonso Cruz-Ramos, Carmen Gahia Facundo-Medina, Susana Del Toro-Arreola, Miriam Ruth Bueno-Topete

**Affiliations:** ^1^ Departamento de Biología Molecular y Genómica, Instituto de Investigación en Enfermedades Crónico Degenerativas, Centro Universitario de Ciencias de la Salud, Universidad de Guadalajara, Guadalajara, Jalisco, Mexico; ^2^ Laboratorio de Inmunología Traslacional, Departamento de Biología Celular y Molecular, Centro Universitario de Ciencias Biológicas y Agropecuarias, Universidad de Guadalajara, Zapopan, Jalisco, Mexico; ^3^ Coordinación de Investigación, Subdirección de Desarrollo Institucional, Instituto Jalisciense de Cancerología, Guadalajara, Jalisco, Mexico; ^4^ Laboratorio de Inmunología, Departamento de Fisiología, Centro Universitario de Ciencias de la Salud, Universidad de Guadalajara, Guadalajara, Jalisco, Mexico

**Keywords:** gut microbiota, NK cell, dysbiosis, cervical cancer, immune exhaustion, immune checkpoints

## Abstract

Alterations in gut microbiota composition have been implicated in various diseases, including cancer. Recent evidence suggests that intestinal microbiota may influence the efficacy of immunotherapy. In this study, we investigated the relationship between gut dysbiosis and NK cell exhaustion in Mexican patients with cervical cancer (CC), a connection not previously explored. This cross-sectional study included newly diagnosed CC patients, a separate cohort of post-radio-chemotherapy (RCT) patients, and healthy donors (HD). Fecal microbiota profiles were assessed using 16S rRNA sequencing, while peripheral NK cell immune checkpoint expression was analyzed by multiparametric flow cytometry. CC patients exhibited significant gut dysbiosis, marked by reduced α-diversity, enrichment of pro-inflammatory taxa (*Escherichia*-*Shigella*, *Prevotella*), depletion of short-chain fatty acid (SCFA)-producing bacteria (*Ruminococcus*, *Christensenellaceae*), and enrichment of microbial metabolic pathways related to inflammation, oxidative stress, nutrient limitation, and immune suppression. Dysbiosis was more pronounced in patients after RCT, with further enrichment of *Phascolarctobacterium*. In parallel, NK cells displayed a putative exhausted phenotype, with elevated expression and co-expression of PD-1, LAG-3, TIM-3, TIGIT, BTLA, and NKG2A. A dysbiosis score and an NK exhaustion score were developed, revealing a significant positive correlation between microbial imbalance and NK cell exhaustion. Machine learning analysis identified the *Escherichia*/*Ruminococcus* ratio and PD-1^+^CD56^bright^ NK cells as predictive markers of CC. Moreover, both dysbiosis and NK cell exhaustion markers were significantly associated with reduced patient survival. This is the first study to demonstrate a link between gut microbiota alterations and NK cell exhaustion in CC. Our findings suggest that gut dysbiosis may contribute to impaired anti-tumor immunity. This study supports the rationale for microbiota-targeted interventions as adjunctive strategies in CC, although prospective validation is required.

## Introduction

Cervical cancer (CC) remains a major global health burden, particularly in low- and middle-income countries, where it is among the leading causes of cancer-related mortality in women. In Mexico, CC is the second most common cancer and the fourth leading cause of cancer-related death among women. Despite advancements in treatment, including radio-chemotherapy (RCT), survival rates for advanced-stage CC remain suboptimal (1). Recently, the microbiota has emerged as a crucial modulator of cancer development and progression, now recognized as an enabling hallmark of cancer (2).

Among the various microbiomes in the human body, the gut microbiota has gained significant attention due to its systemic influence on distant organs and physiological processes, including immune system regulation. Crosstalk between the gut and vaginal microbiota occurs through vertical transmission and direct translocation of rectal microbes to the vaginal environment (3). Additionally, the gut microbiota can contribute to the modulation of vaginal microbiota through estrogen metabolism. Certain intestinal bacteria produce β-glucuronidases, enzymes that deconjugate estrogens previously metabolized in the liver, facilitating their reabsorption into circulation. This process, carried out by the estrobolome, influences estrogen availability (4), which in turn affects the vaginal microbiota by promoting *Lactobacillus* growth and microbial homeostasis in the vagina (5). However, disruptions in this pathway may alter vaginal microbial diversity, potentially impacting gynecological health, as elevated estrogen levels have been associated with an increased risk of malignancies (6).

Dysbiosis, an imbalance in microbial composition, has been implicated in various diseases, including cancer. Alterations in the gut microbiota can impact immune homeostasis, chronic inflammation, and the efficacy of cancer therapies, highlighting the potential role of the microbiota as both a biomarker and a therapeutic target in oncology (2). Recent studies have reported significant gut microbiota alterations in CC patients, including the enrichment of *Prevotella*, *Porphyromonas*, *Dialister*, *Proteobacteria*, and *Escherichia*-*Shigella*, alongside a depletion of beneficial short-chain fatty acid (SCFA)-producing bacteria such as *Blautia*, *Alistipes*, *Clostridia* and *Ruminococcus* (7–10). Moreover, RCT has been shown to further disrupt microbial diversity, reducing beneficial taxa while promoting the expansion of pro-inflammatory bacteria, including *Proteobacteria*, *Gammaproteobacteria*, and *Haemophilus* (11). Notably, specific microbial signatures characterized by decreased microbial diversity and increased *Escherichia*-*Shigella* and *Enterobacteriaceae* have been associated with poor prognosis and reduced survival in CC patients (12).

The gut microbiota plays a key role in modulating systemic immune responses, including the function of natural killer (NK) cells (13). As frontline effectors of anti-tumor immunity, NK cells are essential for the rapid detection and elimination of malignant cells, a process tightly regulated by the balance between activating and inhibitory signals. However, in CC, NK cells often exhibit an exhausted phenotype, characterized by increased expression of inhibitory molecules, such as PD-1, TIGIT, and TIM-3, leading to impaired cytotoxic activity (14).

The gut microbiota has been shown to influence the response to immune checkpoint blockade (ICB) therapy. Specific gut microbial profiles and greater microbial diversity have been correlated with enhanced responses to PD-1, PD-L1, and CTLA-4 blockade therapies in various cancers, possibly due to microbiota-immune modulation or microbiota-drug interaction (15–18). Beyond therapy response, microorganisms regulate immune function via direct cell–cell interactions (19) and microbial metabolites (20), which under a dysbiotic state could translocate due to compromised integrity of the intestinal barrier and promote immune exhaustion (21).

Despite the growing interest in the interplay between the microbiota and the immune system, most studies have focused on T cell-mediated immunity, leaving the relationship between gut dysbiosis and NK cell exhaustion largely unexplored. RCT remains the standard treatment for locally advanced CC; however, its systemic effects, particularly on immune function and microbiota composition, are not fully understood. Importantly, the therapeutic landscape of CC is evolving beyond conventional RCT. ICB has demonstrated clinical benefit in recurrent and metastatic disease and is increasingly being incorporated into systemic regimens, often in combination with RCT and anti-angiogenic agents. These advances reinforce the importance of host–microbiota interactions in shaping responsiveness to therapeutic combinations that are likely to become standard in the coming years, highlighting the need for deeper investigation into how the microbiota modulates treatment outcomes (22).

To our knowledge, this is the first study in Mexico to characterize the gut microbiota in CC patients and to explore its association with NK cell exhaustion and the effects of standard RCT. This work provides novel insights to the study of host–microbiota–immunity interactions in a Western Mexican cohort.

## Materials and methods

### Approval of clinical research

The recruitment of CC patients was conducted at the Instituto Jalisciense de Cancerología in Guadalajara, Jalisco, Mexico. Clinically healthy female donors (HD) from the community participated as the control group. This study was performed following the ethical principles outlined in the Declaration of Helsinki (2024 revision) and was approved by the Research and Ethics Committees of the health institution (PRO-72/23) as well as by the University Center (22-92-CI-00323). All participants were informed about the objectives of the study, and written informed consent was obtained before their inclusion.

### Study design

This cross-sectional study included 77 female participants: 49 patients diagnosed with cervical cancer (CC) and 28 age-adjusted healthy donors (HD). From each participant, fecal samples were collected for gut microbiota profiling, and peripheral blood was obtained for NK cell phenotypic analysis. CC patients were stratified into two groups: 27 treatment-naïve individuals with newly diagnosed CC, assigned as CC pre-treatment group, and 22 patients who had completed standard-of-care radio-chemotherapy (RCT), which consisted of 50 Gy delivered in 25 fractions with concomitant platinum-based chemotherapy, within the previous two weeks, assigned as CC post-treatment. The control group comprised 28 healthy women with no history of malignancy, confirmed by a negative Papanicolaou test within the past year. Written informed consent was obtained from all participants prior to enrollment.

### Inclusion and exclusion criteria

Inclusion criteria for the CC group were: (i) age ≥18 years, (ii) histopathologically confirmed cervical cancer (stages I–IV, classified by TNM system), and (iii) provision of informed consent. Exclusion criteria for all participants included: (i) use of prebiotics or probiotics within four weeks before recruitment, (ii) any chronic infection other than HPV, (iii) hospitalization within the past three months due to COVID-19-related illness, (iv) diagnosed gastrointestinal disorders, (v) autoimmune diseases and (vi) pregnancy.

### Extraction of nucleic acids and 16S rRNA amplicon sequencing

Fecal samples were collected and immediately stored at −80°C. DNA was extracted from 150 mg of frozen feces with Quick-DNA™ Fecal/Soil Microbe Miniprep (Zymo Research, USA, cat: D6010) according to the manufacturer’s protocol. DNA was quantified using a NanoDrop™ OneC spectrophotometer (Thermo Scientific, Waltham, MA, USA). The 16S metagenomic sequencing library preparation was performed according to the Illumina MiSeq System protocol (Illumina, San Diego, CA, USA) (23). V3 and V4 regions from 16S were amplified with Platinum Taq DNA Polymerase High fidelity (Invitrogen, Waltham, MA, USA) using primers with adaptors. The sequence of the primers used was: Forward: (5’TCGTCGGCAGCGTCAGATGTGTATAAGAGACAGCCTACGGGNGGCWGCAG-3’), reverse: (5’GTCTCGTGGGCTCGGAGATGTGTATAAGAGACAGGACTACHVGGGTATCTAATCC- 3’). PCR conditions were performed according to the Illumina protocol. Product purification was achieved using AMPure XP^®^ (Beckman Coulter, Indianapolis, IN, USA) magnetic beads and was quantified with the Qubit^®^ 3 dsDNA HS kit (Invitrogen, Waltham, MA, USA) according to product indications. Next, index incorporation was achieved with the Nextera XT Index Kit v2 Set A (No. Cat. FC-131-2001, Illumina, San Diego, CA, USA) by a second PCR amplification. Finally, amplicons were pooled to equimolar concentrations into a 4 nmol/L solution tube, which were then denatured, and was further diluted to the recommended loading concentration for the MiSeq Sample Loading (kit Miseq Reagent V3 600-cycle, Illumina, San Diego, CA, USA). The sequencing was performed according to the manufacturer´s protocol.

### Bioinformatic analysis

Analysis of 16S rRNA (V3-V4) sequences was performed using QIIME2 version 2024.2 amplicon distribution (24). Previously, the sequences whose Phred score were higher than 30 were processed. Then, raw reads (at least 100, 000 raw reads per sample) were further denoised using DADA2 via *q2-dada2* (25) at default settings. Analyses were conducted with an average of 40, 000 denoised amplicon sequence variants (ASVs) per sample. Taxonomy assignation of our sequences was performed using a full-length 16S trained classifier (26), further employing Silva 138.1 as a reference taxonomic database (27, 28). ASVs identified as mitochondria and chloroplasts were removed. Then, filtered ASVs were aligned using multiple alignment using fast Fourier transform (MAFFT) via *q2-alignment*, and phylogeny was built with FastTree2 via *q2-phylogeny* (29). A-diversity indices (30–33) and β-diversity distances (Bray-Curtis, Jaccard, unweighted and weighted Unifrac) (34, 35) were computed via *q2-diversity*. Principal coordinate analysis (PCoA) plots were generated to visualize β-diversity distances using Emperor via *q2-emperor* (36) and the different distances were further analyzed using permutational multivariate analysis of variance (PERMANOVA) tests.

Differential abundance analyses at the genus level were performed using analysis of compositions of microbiomes with bias correction (ANCOM-BC) via *q2-composition* (37). Before analysis, a frequency filter was applied in which features that appeared more than 50 times in at least 10% of the samples were retained. A *q ≤* 0.05 cut-off was used to assess significance, and a log fold change (LFC) ≥|1.0| to evaluate the effect size. To assess the potential metabolic profile of the gut microbiota, phylogenetic investigation of communities by reconstruction of unobserved states 2 (PICRUSt2) pipeline (38–42) was employed, coupled with the MetaCyc Database (43). The resulting pathways were further analyzed using ANCOM-BC, using the previously described parameters. Different taxonomic ratios were calculated following previously published methods (44).

### Flow cytometry

Peripheral blood samples were collected using 10 mL K2-EDTA spray-coated tubes (Becton Dickinson, Franklin Lakes, New Jersey, USA; 366643) for the separation of peripheral blood mononuclear cells (PBMCs). PBMCs were isolated via density gradient centrifugation using Lymphoprep (Stemcell Technologies, Vancouver, British Columbia, Canada; 07851).

After isolation, PBMCs were stored in liquid nitrogen at −196°C until further analysis. Cell viability was assessed using trypan blue exclusion, and only samples exhibiting ≥90% viability were included in the following analyses.

A multi-parametric flow cytometry panel was employed to evaluate the expression of immune checkpoint molecules (PD-1, TIGIT, NKG2A, BTLA, TIM-3, and LAG-3) on NK cell subsets. Viability was evaluated in every sample in an independent staining using Zombie NIR™, and every sample showed a viability higher than 90%. The following monoclonal antibodies were utilized for staining 5 × 10^5^ PBMCs: Anti-CD3-FITC [fluorescein isothiocyanate, 300406], Anti-CD56-BV711 [Brilliant Violet 711, 362542], Anti-CD16-BV605 [Brilliant Violet 605, 302040], anti-CD45-AF700 [Alexa Fluor 700, 304024), Anti-TIGIT- PE [phycoerythrin, 372704], Anti-TIM-3-BV510 [Brilliant Violet 510, 345030], Anti-PD-1-BV421 [Brilliant Violet 421, 329920], Anti-LAG-3-PerCP/Cy5.5 [peridinin chlorophyll/cyanine 5.·5, 369312], Anti-BTLA-PE/Cy7 [phycoerythrin/cyanine 7, 344516], Anti-NKG2A-APC [allophycocyanin, 375108]. All antibodies were sourced from BioLegend (San Diego, CA, USA). Data acquisition was conducted on an Attune™ NxT Flow Cytometer (Thermo Fisher Scientific, Waltham, MA, USA). Compensation was made with compensation beads (Becton Dickinson, Franklin Lakes, NJ, USA; 55284). For each sample, singlet cells were identified using forward scatter-area (FSC-A) *vs.* forward scatter-height (FSC-H) dot plots, followed by gating lymphocytes using FSC-A *vs.* side scatter-area (SSC-A) plots. A total of 250, 000 events were recorded within the lymphocyte gate. Single and co-expression of immune checkpoint receptors was analyzed in peripheral NK cell populations using Kaluza software (version 2.1, Beckman Coulter, Brea, CA, USA). PBMCs were analyzed as CD3^-^CD56^dim^ and CD3^-^CD56^bright^ NK cell populations.

### Calculation of exhaustion and dysbiosis scores

To standardize NK cell phenotyping and provide an integrative measure of exhaustion, we developed a composite Global NK Exhaustion Score. For each inhibitory receptor (PD-1, LAG-3, BTLA, TIM-3, TIGIT, NKG2A), Z-scores were calculated relative to the healthy donor distribution. The calculated Z-scores were then classified by specified percentiles, as explained in **Supplementary Material 1**, to obtain a score (1 to 3). These values were summed separately for CD56^dim^ and CD56^bright^ NK subsets. The Global NK Exhaustion Score for each patient was defined as the mean of the two subset-specific scores (CD56^dim^ and CD56^bright^), yielding a composite index of inhibitory receptor burden. This approach was inspired by previous work in T cells, where transcriptomic-based exhaustion scores were generated by integrating multiple exhaustion-related features into a unified index (45). Conceptually, our strategy also reflects the notion that NK dysfunction cannot be defined by a single marker but rather emerges from the combined expression of multiple inhibitory receptors and phenotypic alterations (46). Together, this percentile-based score provides a standardized framework to compare NK exhaustion across patients and to correlate it with microbiota alterations.

The microbiota dysbiosis score included α-diversity metrics (Shannon, Pielou, Simpson, and Strong indices) and the centered log-ratio (CLR)-transformed relative abundances of bacterial taxa previously identified as expanded or depleted in CC patients by ANCOM-BC analyses. After Z-score calculation, each parameter was scored using a similar percentile-based classification scheme, capturing deviations in either direction from control values, as both increases and decreases may reflect dysbiosis. Finally, correlation analysis between NK exhaustion and microbiota dysbiosis scores was performed. Detailed formulas and score calculation workflows are provided in **Supplementary Material 1**.

### Statistical analyses

Data distribution was assessed for normality using the D’Agostino–Pearson normality test. For comparisons between two groups, parametric data were analyzed using the Student’s T-test, while non-parametric data were analyzed using the Mann–Whitney U test. For comparisons involving three or more groups, a one-way analysis of variance (ANOVA) was applied for parametric data, with *post-hoc* comparisons adjusted using the Benjamini-Hochberg false discovery rate (FDR) method. The Kruskal-Wallis test with the FDR method of Benjamini-Hochberg for multiple comparisons was used for non-parametric data.

Microbiota composition and diversity analyses were conducted using non-parametric tests (Kruskal-Wallis) within the QIIME2 package. Correlation between microbiota and NK cell exhaustion markers was assessed using Spearman’s rank correlation coefficient.

Sociodemographic data were analyzed using the Chi-square test and are presented as frequencies and percentages. Statistical analyses were performed using GraphPad Prism, version 10.4. and R Studio version 4.4.2. P-values ≤ 0.05 were considered statistically significant.

### Machine learning analysis

A Random Forest (RF)-based classification model was developed to predict CC using microbiota composition, NK cell exhaustion markers, and patient demographic data. The dataset consisted of 104 variables, including microbial genera abundances, diversity indices, NK-cell receptor expression profiles, along with clinical variables such as age and BMI. Only HD and newly diagnosed, pre-treatment CC patients were considered for analysis. This approach was adapted from Tsakmaklis et al., 2023 (47). Briefly, feature selection was performed using Recursive Feature Elimination (RFE) with leave-group-out (Monte-Carlo) cross-validation (1000 iterations) to minimize overfitting and retain the 20 most predictive variables. The final RF model was trained with 200 decision trees (ntree=200) and an empirically determined mtry of 2. Model performance was assessed through repeated random partitioning (1000 iterations) into training (70%) and test (30%) subsets. In each iteration, the model was independently trained and tested, and its predictive ability was evaluated using the area under the receiver operating characteristic curve (ROC-AUC), reported as the mean and standard deviation across iterations reported as the mean and standard deviation (SD) across iterations. The SD represents the variability of model performance across these repeated cross-validation runs, providing an estimate of the model’s stability and generalizability. Classification accuracy was assessed using a confusion matrix, calculating sensitivity, specificity, and overall accuracy. To further validate the model and reduce predictor variables, stepwise logistic regression was applied to the most important features identified through RF analysis. The final logistic regression model was developed with the selected variables and assessed for its predictive accuracy.

To assess the impact of microbiota and NK cell exhaustion markers on patient survival, an independent mortality prediction model was developed, which was restricted to pre-treatment CC patients using a 106-variable data set. The XGBoost (Extreme Gradient Boosting) model demonstrated superior performance to RF and was selected as the final model, which was trained using 10-fold cross-validation with hyperparameter optimization through grid search. Performance was assessed through 1000 iterations of random partitioning (70% training, 30% testing). Similarly, stepwise logistic regression was applied to the most important predictors identified by XGBoost to refine the model and assess its predictive power.

For each selected variable, Kaplan-Meier survival curves were generated to assess the differences in survival between groups. The cutoff value for categorizing patients into “high” and “low” groups was determined by the point of maximum sensitivity and specificity on a ROC curve for each variable. This approach allowed a distinction between patients who survived and those who did not. Subsequently, Kaplan-Meier curves were constructed for survival analysis of the CC pre-treatment patients over the 15-month follow-up period.

## Results

### Cross-sectional study and characteristics of participants

The Clinicopathological characteristics of the study participants are presented in **Table 1**. No significant differences were observed in the age across groups (*p* = 0.9658). However, BMI was significantly higher in CC pre-treatment patients compared to HD (*p* = 0.0238), with a greater prevalence of patients classified as overweight or with obesity. The most common histological type was squamous cell carcinoma, accounting for most cases in both the pre- and post-treatment groups. Stool consistency, assessed by the Bristol Scale, shifted from a median of 4 (IQR: 3–4) in HD to 3 (IQR: 2–6) in CC pre-treatment and 5 (IQR: 4–6) in post-treatment patients, consistent with bowel habit alterations linked to disease and treatment. All post-treatment patients underwent a standard regimen of fractionated radio-chemotherapy (RCT), receiving a total dose of 50 Grays in 25 sessions, concomitant with platinum-based chemotherapy.

**Table 1:** Demographical and clinical characteristics of study groups.

Variable	HD (*n*=28)	CC pre-tx (*n*=27)	CC post-tx (*n*=22)	*P* value
Age (years, mean ± SD)	41.75 ± 12.09	41.52 ± 13.05	42.41 ± 10.63	0.9658^†^
BMI (kg/m^2^, mean ± SD)	23.83 ± 3.40^b^	27.26 ± 5.09^a^	25.87 ± 6.54	0.0276*^‡^
Underweight (n, %)	0 (0%)	1 (3.7%)	1 (4.5%)	0.0356*^§^
Normal weight (n, %)	18 (64.3%)	8 (29.6%)	10 (45.5%)	
Overweight (n, %)	10 (35.7%)	12 (44.4%)	9 (40.9%)	
Obesity (n, %)	0 (0%)	6 (22.2%)	2 (9.1%)	
Bristol scale (median [IQR])	4 [3-4]	3 [2-6]	5 [4-6]	0.1586^†^
Clinical stage (n, %)				0.1107^§^
I		10 (37%)	2 (9.1%)	
II		7 (25.9%)	6 (27.3%)	
III		9 (33.3%)	12 (54.5%)	
IV		1 (3.7%)	2 (9.1%)	
Histology (n, %)				0.1854^§^
Epidermoid		23 (85.2%)	15 (68.2%)	
Adenocarcinome		4 (14.8%)	7 (31.8%)	

Data are expressed as mean ± SD, median [IQR], or n (%). Differences between groups were assessed using: ^†^One-way ANOVA; ^‡^Kruskal–Wallis; ^§^Fisher’s exact test. *Post-hoc* pairwise comparisons, when applicable, were adjusted with the Benjamini–Hochberg FDR method. Significant differences between groups are indicated by superscript letters (a=HD; b=CC pre-tx; c=CC post-tx). HD, healthy donors; CC pre-tx, cervical cancer patients before treatment; CC post-tx, cervical cancer patients after treatment; SD, standard deviation; IQR, interquartile range; BMI, body mass index. A *p*-value<0.05 was considered statistically significant.

### Disruption of gut microbiota diversity in newly diagnosed cervical cancer patients and cancer patients after radio-chemotherapy

**Figure 1:**
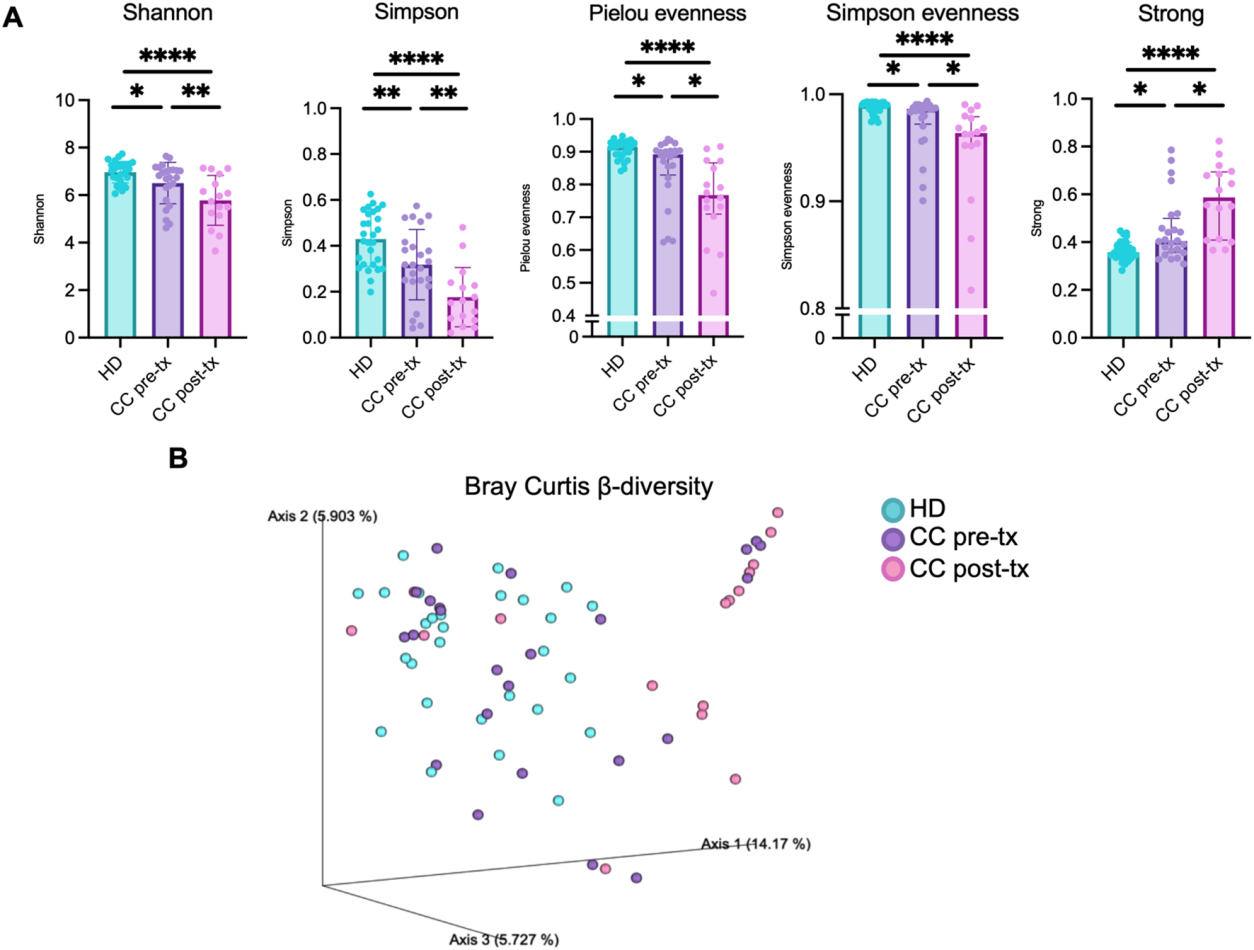
Gut microbiota diversity in fecal samples from healthy donors (HD), CC patients before treatment (CC pre-tx), and CC patients after treatment (CC post-tx). **(A)** α-diversity metrics: Shannon, Simpson, Pielou evenness, Simpson evenness and Strong. Comparisons between groups were performed using one-way ANOVA for parametric data (Shannon and Simpson), and the Kruskal-Wallis test for non-parametric data (Pielou evenness, Simpson evenness, and Strong), followed by the Benjamini-Hochberg FDR method for multiple comparison correction. Data are shown as mean ± SD for parametric variables and median with IQR for non-parametric variables. **(B)** β-diversity: Three-dimensional scatter plot obtained by PCoA using the Bray-Curtis distance, showing the distance between study groups in terms of β-diversity. Statistical analyses were performed using PERMANOVA to determine the statistical significance of the observed separations in the coordinate space. *p ≤ 0.05, **p≤ 0.01, ****p ≤0.0001. FDR, False Discovery Rate; PCoA, Principal Coordinates Analysis; PERMANOVA, Permutational Multivariate Analysis of Variance.

The α-diversity of the gut microbiota was evaluated using Shannon, Simpson, Pielou evenness, Simpson evenness, and Strong metrics (**Figure 1A**). All comparisons showed significant differences between groups (**Supplementary Table 2A**). CC patients exhibited a marked reduction in overall diversity compared with HD, which was further aggravated after RCT. This reduction reflected both a loss of diversity (Shannon, Simpson) and evenness (Pielou, Simpson evenness), alongside increased bacterial dominance (Strong). This shift indicates a profound imbalance in gut microbiota, characterized by the overrepresentation of specific taxa that may contribute to chronic inflammation and dysbiosis. RCT further exacerbated this disruption, amplifying bacterial dominance, intensifying the loss of microbial diversity and homeostasis in CC patients.

β-diversity was assessed using Bray–Curtis (**Figure 1B**), Jaccard, weighted, and unweighted UniFrac metrics (**Supplementary Figure 2B**), and visualized through Principal Coordinate Analysis (PCoA). HD samples formed a relatively tight cluster, while pre-treatment CC samples displayed a broader distribution, indicating greater inter-individual variability. In contrast, post-treatment CC samples were more distantly separated from HD and clustered more compactly, reflecting a pronounced shift in microbial community structure. PERMANOVA confirmed significant differences between groups (**Supplementary Table 2C**), underscoring the strong disruption of gut microbiota composition in CC patients, which was further exacerbated following RCT.

Additionally, we assessed gut microbiota alpha and beta diversity in CC patients stratified by clinical stage, both before and after treatment. However, no significant compositional differences were observed across stages, and further analyses of microbiota-related parameters were not pursued (not shown).

### Shift in relative abundance of gut microbiota in newly diagnosed cervical cancer patients and cancer patients after radio-chemotherapy

**Figure 2:**
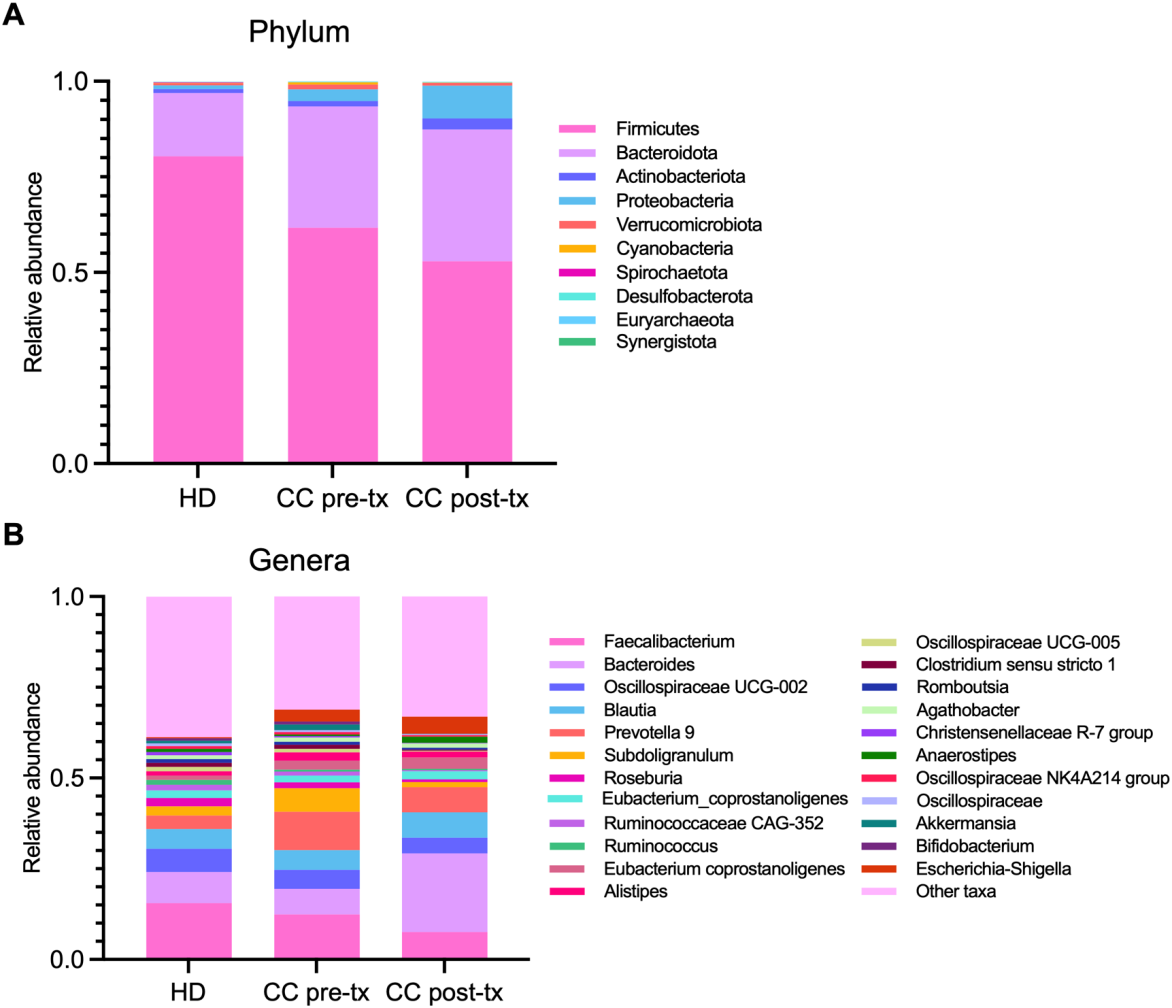
Relative abundance of gut microbiota in fecal samples from healthy donors (HD), CC patients before treatment (CC pre-tx), and CC patients after treatment (CC post-tx). **(A)** Phylum-level and **(B)** genus-level relative abundances are represented as stacked bar plots of ASVs detected in the study groups. ASVs, Amplicon Sequence Variants.

At the phylum level, pre-treatment CC patients displayed a reduction in the abundance of *Firmicutes* (80.35% *vs.* 61.66%), an enrichment of *Bacteroidota* (16.53% *vs.* 31.72%) and *Proteobacteria* (0.90% *vs.* 3.04%) in comparison to HD. This shift was even more pronounced in post-treatment patients (*Firmicutes* 52.92%, *Bacteroidota* 34.48%, *Proteobacteria* 8.52%), with a further expansion of *Actinobacteriota* (1.08% *vs.* 2.91%) (**Figure 2A**).

At the genus level, pre-treatment CC patients showed an expansion of pathobionts, including *Prevotella* (3.72% *vs.* 10.55%) and *Escherichia-Shigella* (0.29% *vs.* 3.29%), alongside a decrease in SCFA-producing bacteria such as *Faecalibacterium* (15.53% *vs.* 12.43%), *Ruminococcus* (1.35% *vs.* 0.53%), and *Roseburia* (2.31% *vs.* 1.64%) compared to HD. Post-treatment patients exhibited even greater enrichment of *Escherichia-Shigella* (4.68%), accompanied by an increase in *Bacteroides* (8.56% *vs.* 21.67%) and *Anaerostipes* (0.85% *vs.* 1.73%), and pronounced reduction in *Faecalibacterium* (7.55%), *Ruminococcus* (0.30%) *Roseburia* (0.78%), *Akkermansia* (0.72% *vs.* 0.09%), and *Subdolingranulum* (2.50% *vs.* 1.42%), in contrast to HD (**Figure 2B**).

### Key bacteria associated with newly diagnosed cervical cancer patients and cancer patients after radio-chemotherapy

**Figure 3:**
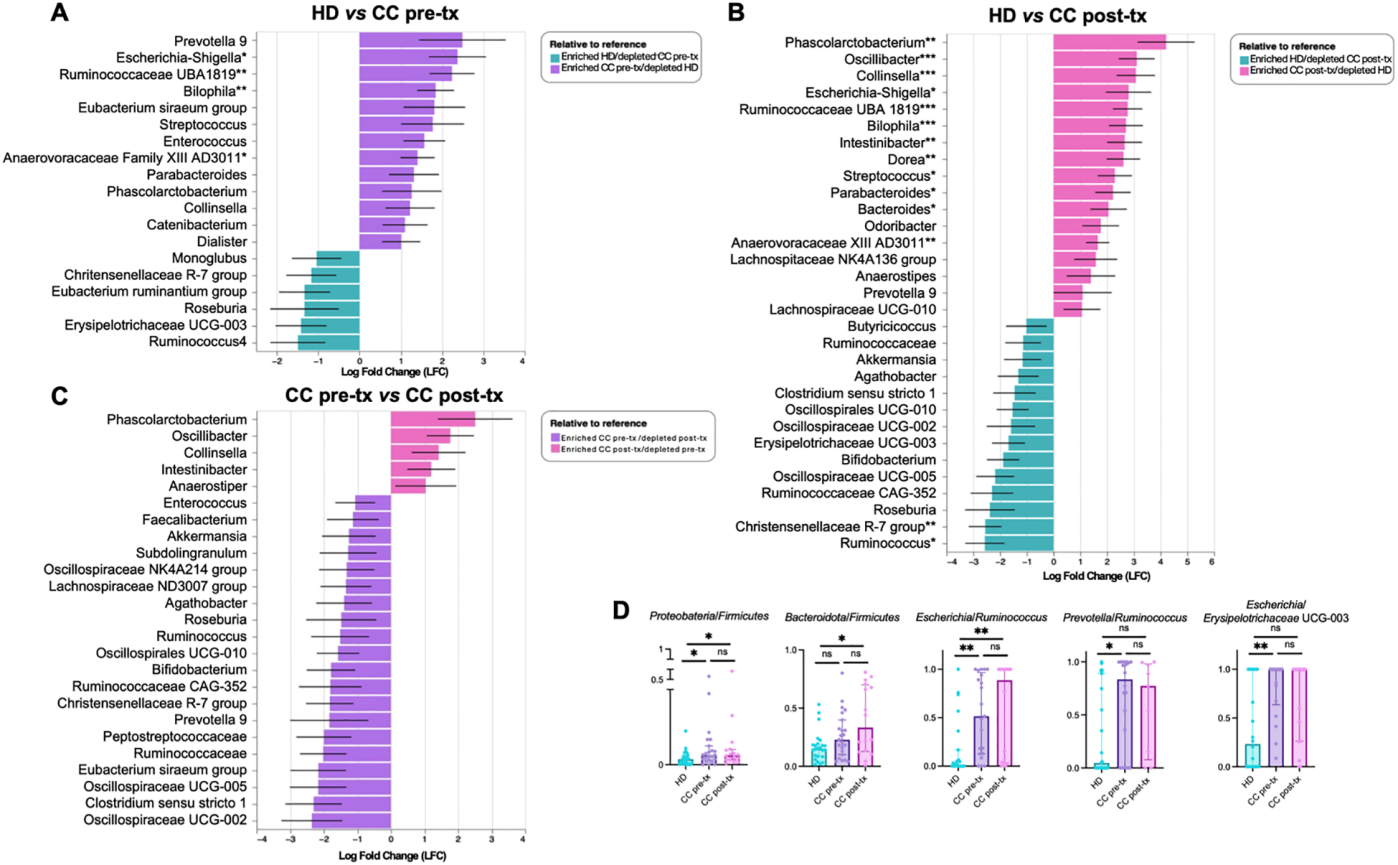
Differential taxon analysis of gut microbiota in fecal samples from healthy donors (HD), CC patients before treatment (CC pre-tx), and CC patients after treatment (CC post-tx). **(A)** Comparison of HD vs. CC pre-tx, **(B)** Comparison of HD vs. CC post-tx, and **(C)** Comparison of CC pre-tx vs. CC post-tx at the family and genus levels using ANCOM-BC. Blue bars represent bacterial taxa enriched in HD, purple bars in CC pre-tx, and pink bars in CC post-tx. Bars indicate the LFC obtained by ANCOM-BC between study groups, using an LFC cutoff of ±1.5. ANCOM-BC. **(D)** Ratios of CLR-transformed relative abundance of selected taxa between groups. Comparisons between groups were performed using the Kruskal-Wallis test for non-parametric data, followed by the Benjamini-Hochberg FDR method for multiple comparison correction. Data are shown as median with IQR. *p ≤ 0.05, **p ≤ 0.01, ***p ≤ 0.001. LFC, Log Fold Change; ANCOM-BC, Analysis of Compositions of Microbiomes with Bias Correction; CLR, Centered Log-Ratio.

The ANCOM-BC analysis identified statistically significant microbial taxonomic shifts between pre-treatment, post-treatment, and HD groups, providing insights into the key bacteria associated with the disease and treatment. In pre-treatment patients, when compared to HD, *Prevotella* 9 showed the highest enrichment (LFC = 2.49) and emerged as a hallmark species of this group. This was accompanied by an increased abundance of several other microbial taxa, notably within the *Proteobacteria* phylum, including *Escherichia-Shigella* (*p* = 0.0319) and *Bilophila* (*p* = 0.0019). Additionally, significant enrichment was observed in several bacteria within the *Firmicutes* phylum, including *Streptococcus*, *Enterococcus*, *Ruminococcaceae* UBA 1819 (*p* = 0.0020), *Parabacteroides*, *Phascolarctobacterium*, and *Anaerovoracaceae* XII AD3011 (*p* = 0.0360) (**Figure 3A**).

Post-treatment patients exhibited a marked alteration in their microbial communities. While some similarities to the pre-treatment microbiota were observed, post-treatment individuals demonstrated a more pronounced enrichment of inflammation-associated bacteria. Compared to HD, key bacteria with significant increase included *Phascolarctobacterium* (*p* = 0.0039), *Escherichia-Shigella* (*p* = 0.0400), *Streptococcus* (*p* = 0.0168), *Bilophila* (*p* = 0.0009), *Parabacteroides* (*p* = 0.0351), and *Anaerovoracaceae* XII AD3011 (*p* = 0.0073). Compared to pre-treatment, there was a particular enrichment of *Phascolarctobacterium* (LFC 4.20), along with *Oscillibacter*, *Collinsella*, and *Intestinibacter*, suggesting potential microbial signatures associated with the therapeutic intervention (**Figure 3B**). Additionally, post-treatment patients exhibited a pronounced depletion of short-chain fatty acid (SCFA)-producing bacteria, including *Ruminococcus* (*p* = 0.0206), *Christensenellaceae* R-7 group (*p* = 0.0015), *Akkermansia*, *Oscillospiraceae*, *Lachnospiraceae*, *Eubacterium*, and *Clostridium* (**Figure 3C**). These results suggest a shift towards a pro-inflammatory microbial profile in the post-treatment state.

The HD group was predominantly characterized by members of the *Firmicutes* phylum such as *Ruminococcus*, *Christensenellaceae* R-7 group, *Eubacterium ruminantium*, *Roseburia*, and genera such as *Bifidobacterium* and *Akkermansia*, which are associated with intestinal health.

Overall, CC patients are characterized by *Prevotella* and *Escherichia*-*Shigella*, while treatment led to the enrichment of specific bacteria, particularly *Phascolarctobacterium*, *Oscillibacter*, *Collinsella*, and *Intestinibacter*, while SCFA-associated taxa, including *Ruminococcus*, *Akkermansia*, *Oscillospiraceae*, and *Lachnospiraceae*, were depleted.

Following these results, a ratio analysis was performed using CLR-transfomed relative abundance of representative taxa (**Figure 3D**) (**Supplementary Material 3**, for ratio calculation), such as *Proteobacteria*/*Firmicutes* (HD *vs.* CC pre- treatment: *p* = 0.0428; HD *vs.* CC post- treatment: *p* = 0.050; CC pre- treatment *vs.* CC post- treatment: *p* = 0.9332), *Bacteroidota*/*Firmicutes* (HD *vs.* CC pre-treatment: *p* = 0.0781; HD *vs.* CC post-treatment: *p* = 0.0112; CC pre-treatment *vs.* CC post-treatment: *p* = 0.3501), *Escherichia*/*Ruminococcus* (HD *vs.* CC pre-treatment: *p* = 0.002; HD *vs.* CC post-treatment: *p* = 0.0017; CC pre-treatment *vs.* CC post-treatment: *p* = 0.7831), *Prevotella*/*Ruminococcus* (HD *vs.* CC pre-treatment: *p* = 0.0215; HD *vs.* CC post-treatment: *p* = 0.1446; CC pre-treatment *vs.* CC post-treatment: *p* = 0.7777), *Escherichia*/*Erysipelotrichaceae* UCG-003 (HD *vs.* CC pre-treatment: *p* = 0.0048; HD *vs.* CC post-treatment: *p* = 0.0615; CC pre-treatment *vs.* CC post-treatment: *p* = 0.4137).

### Functional metagenomic profiles in newly diagnosed cervical cancer patients and cancer patients after radio-chemotherapy

**Figure 4:**
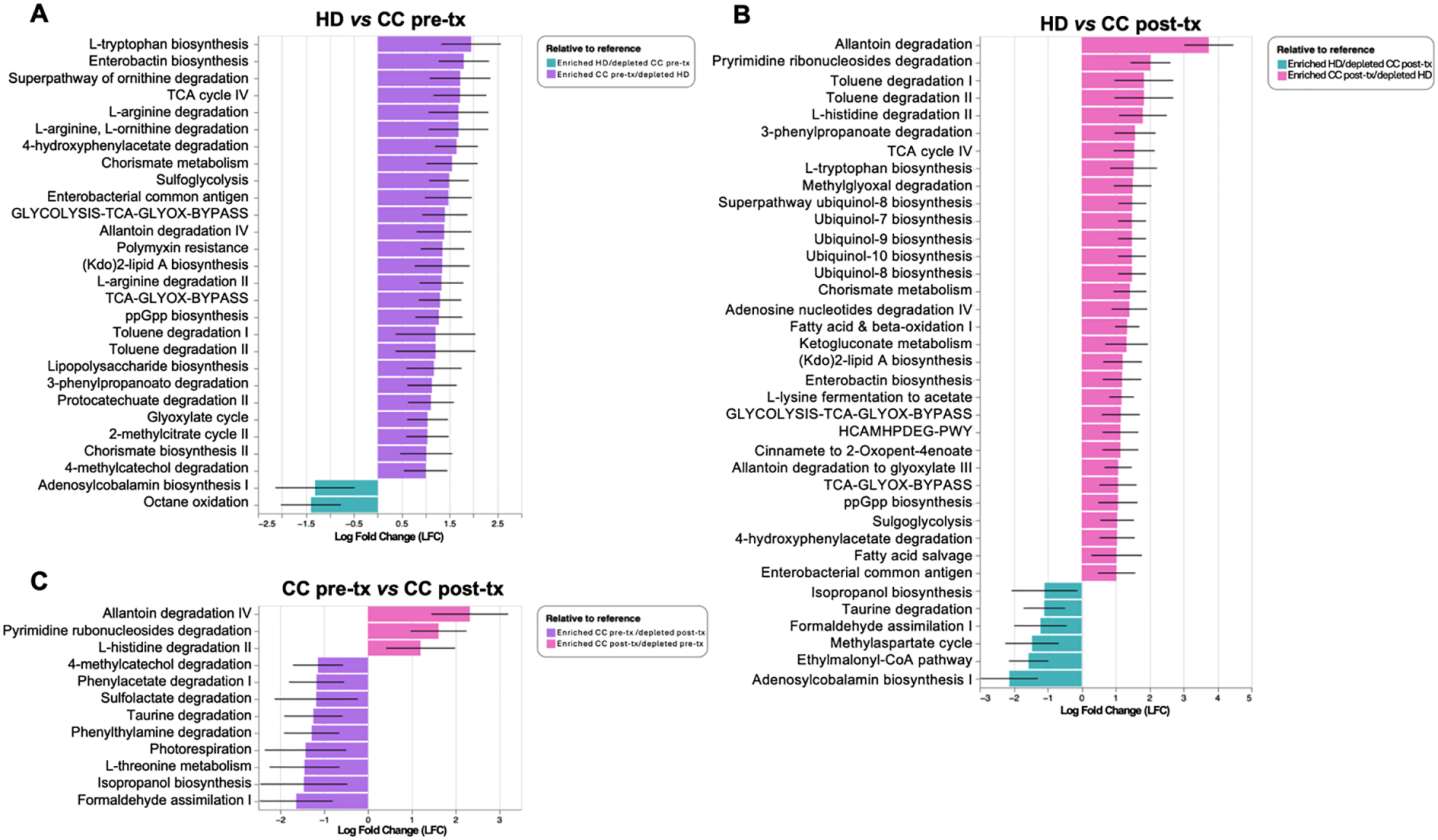
Predictive analysis of metabolic pathways in gut microbiota from fecal samples of healthy donors (HD), CC patients before treatment (CC pre-tx), and CC patients after treatment (CC post-tx). **(A)** Comparison of HD *vs.* CC pre-tx, **(B)** Comparison of HD *vs.* CC post-tx, and **(C)** Comparison of CC pre-tx *vs.* CC post-tx. Blue bars represent metabolic pathways enriched in HD, purple bars in CC pre-tx, and pink bars in CC post-tx. Bars indicate the LFC obtained by PICRUSt2 analysis between study groups, using an LFC cutoff of ±1.LFC, Log Fold Change; PICRUSt2, Phylogenetic Investigation of Communities by Reconstruction of Unobserved States 2.

PICRUSt2 analysis showed that pre-treatment patients exhibit an enrichment of amino acid biosynthesis and metabolism (L-tryptophan biosynthesis, ornithine degradation, L-arginine degradation, L-ornithine degradation, chorismate metabolism, arginine degradation II, and chorismite biosynthesis II), degradation of aromatic compounds and xenobiotics (4-hydroxyphenylacetate degradation, toluene degradation I and II, 3-phenylpropanoate degradation, protocatechuate degradation II, and 4-methylcatechol degradation), inflammation process (enterobactin biosynthesis, enterobacterial common antigen, Kdo2-lipid A and LPS biosynthesis) bacterial stress (ppGpp biosynthesis), antibiotic resistance (polymyxin resistance) and pathways involved in energy metabolism (Glycolysis-TCA-GLYOX-Bypass, TCA-GLYOX-Bypass, sulfoglycolysis, glyoxylate cycle). In contrast, the HD group present an enrichment of adenosylcobalamin biosynthesis and octane oxidation (**Figure 4A**).

Post-treatment patients, in comparison with HD exhibit enrichment of antioxidant-related pathways (ubiquinol-8, -7, -9, and -10 biosynthesis), pathways involved in energy metabolism (TCA cycle IV, Glycolysis-TCA-GLYOX-Bypass, Fatty acid and beta-oxidation), amino acid metabolism (L-tryptophan biosynthesis, and L-histidine degradation II), degradation of aromatic compounds and xenobiotics (toluene degradation I and II, 3-phenylpropanoate degradation, and 4-hydroxyphenylacetate degradation), inflammation-related pathways (enterobactin biosynthesis, enterobacterial common antigen biosynthesis, and Kdo2-lipid A biosynthesis), bacterial stress response (ppGpp biosynthesis), and antibiotic resistance (polymyxin resistance). In contrast, the HD group presented an enrichment of adenosylcobalamin biosynthesis I, ethylmalonyl-CoA pathway, methyl aspartate cycle, formaldehyde assimilation I, taurine degradation, isopropanol biosynthesis (**Figure 4B**).

Comparing pre- and post-treatment patients, pre-treatment showed an enrichment of pathways involved in xenobiotic degradation and detoxification (formaldehyde assimilation I, isopropanol biosynthesis, and sulfolactate degradation), and pathways related to amino acid metabolism (L-threonine metabolism, taurine degradation, and phenylwthylamine degradation), other pathways such as phenylacetate degradation I, along with methylcatechol degradation II, were also observed. In contrast, in the post-treatment group, enrichment of degradation of nucleotide derivatives (allantoin degradation IV and pyrimidine ribonucleosides degradation) and L-histidine degradation II were observed (**Figure 4C**).

### Upregulated immune checkpoint expression in peripheral NK cells of newly diagnosed cervical cancer patients and cancer patients after radio-chemotherapy

**Figure 5:**
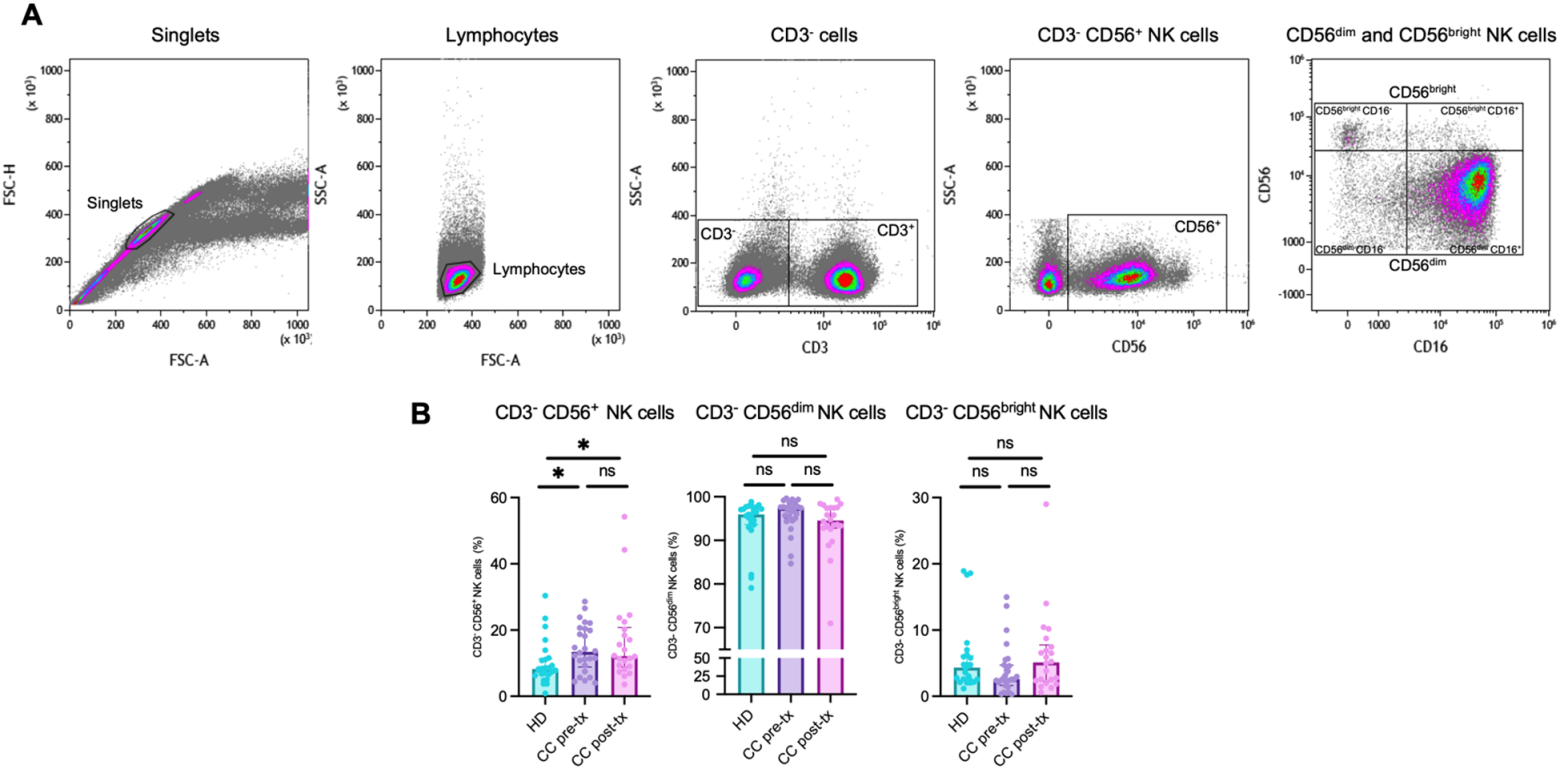
Flow cytometry analysis of NK cell populations in peripheral blood from healthy donors (HD), CC patients before treatment (CC pre-tx), and CC after antineoplastic treatment (CC post-tx). **(A)** Flow cytometry analysis strategy for NK cell populations. **(B)** Percentages of total NK cells (CD3^-^CD56^+^), CD3^-^CD56^dim^ NK cells, and CD3^-^CD56^bright^ NK cells in peripheral blood across the study groups. Comparisons between groups were performed using the Kruskal-Wallis test for non-parametric data, followed by the Benjamini-Hochberg FDR method for multiple comparison correction. Frequency data are presented as individual expression percentages and median with IQR. *p ≤ 0.05.

An initial frequency analysis of NK cell populations (**Figure 5A**) revealed a significant increase in total NK cell frequency in both patient groups compared to healthy women (HD *vs.* CC pre-treatment: *p* = 0.014, HD *vs.* CC post-treatment: *p* = 0.015). In the pre-treatment group, there was a trend toward an expansion of the CD56^dim^ NK cell subset, although this increase did not reach statistical significance (HD *vs.* CC pre-treatment: *p* = 0.1312), accompanied by a decrease in the CD56^bright^ NK cell population (HD *vs.* CC pre-treatment: *p* = 0.1299). Conversely, the post-treatment group exhibited the opposite trend, with a reduction in the CD56^dim^ population (HD *vs.* CC post-treatment: *p* = 0.6263) and a concomitant increase in the CD56^bright^ NK cell subset (HD *vs.* CC post-treatment: *p* = 0.7816). Notably, a direct comparison between pre- and post-treatment patients revealed a near-significant difference in the frequency of both CD56^dim^ (*p* = 0.0508) and CD56^bright^ NK cell subsets (*p* = 0.0809) (**Figure 5B**). NK cells were further subdivided into four subsets based on CD56 and CD16 expression: CD56^dim^CD16^+^, CD56^dim^CD16^-^, CD56^bright^CD16^+^, and CD56^bright^CD16^-^ (48). Among these, we observed a significant expansion of the CD56^bright^CD16^+^ population in post-treatment patients compared with both HD (*p* = 0.0239) and CC pre-treatment groups (*p* = 0.0370). In contrast, the frequencies of the other subsets did not show significant differences across groups (**Supplementary Figure 4**). Analyses of immune checkpoint expression were conducted only on the classical CD56^dim^ and CD56^bright^ NK cell subsets.

**Figure 6:**
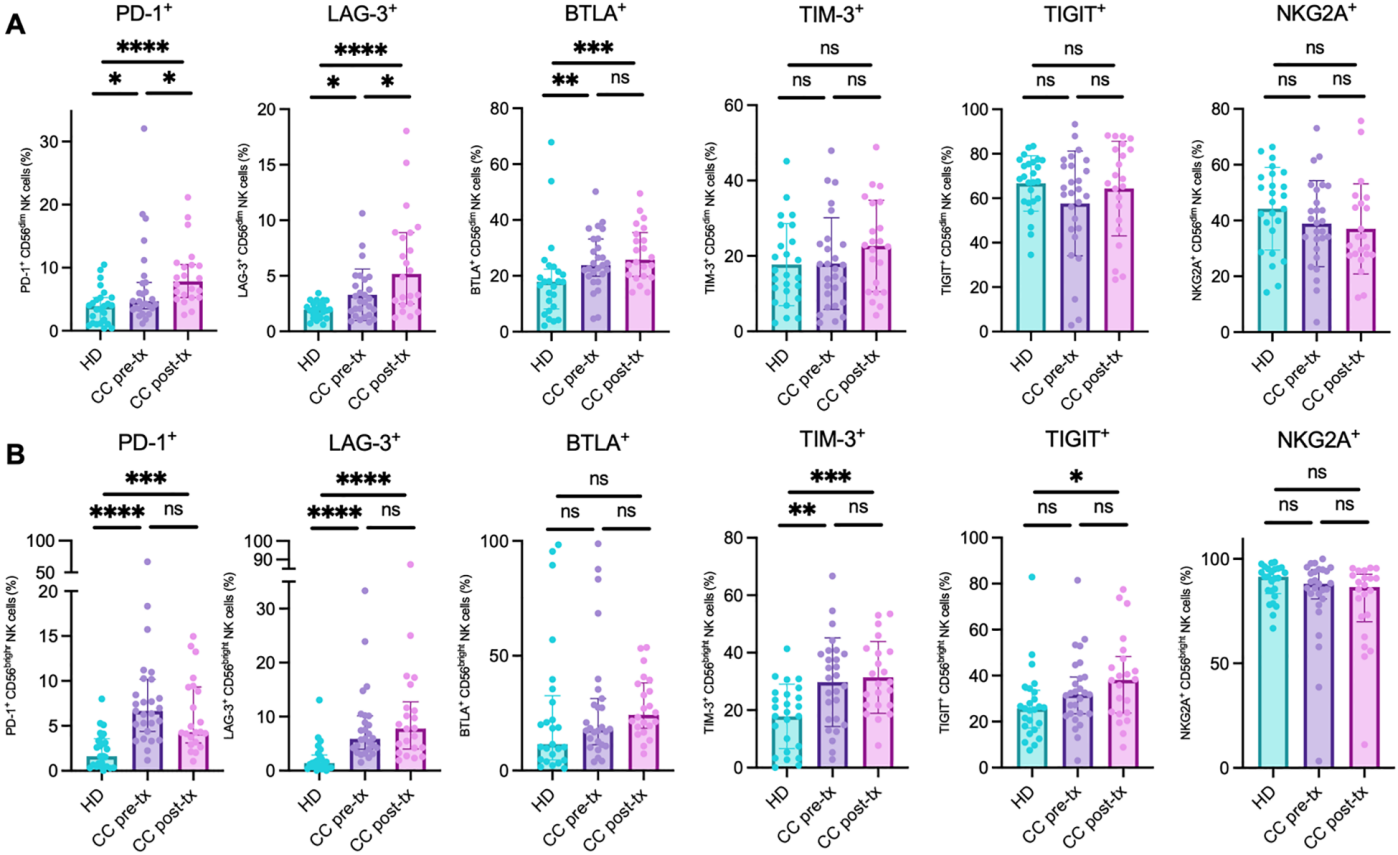
Analysis of immune checkpoint marker analysis in NK cell populations from peripheral blood of healthy donors (HD), CC patients before treatment (CC pre-tx), and CC patients after treatment (CC post-tx). **(A)** Percentage of peripheral CD56^dim^ NK cells expressing inhibitory immune checkpoints. **(B)** Percentage of peripheral CD56^bright^ NK cells expressing immune checkpoints: PD-1, LAG-3, BTLA, TIM-3, TIGIT, and NKG2A. Comparisons between groups were performed using one-way ANOVA for parametric variables: TIM-3 CD56^dim^ and CD56^bright^, TIGIT CD56^dim^, and NKG2A CD56^dim^. Kruskal-Wallis was applied for non-parametric variables: PD-1 CD56^dim^ and CD56^bright^, LAG-3 CD56^dim^ and CD56^bright^, BTLA CD56^dim^ and CD56^bright^, TIGIT CD56^bright^, and NKG2A CD56^bright^. All tests were corrected for multiple comparisons using the Benjamini–Hochberg FDR method. Data are shown as individual percentages of cells expressing each receptor as mean ± SD for parametric variables and median with IQR for non-parametric variables. *p ≤ 0.05, **p≤ 0.01, ***p ≤0.001, ****p ≤0.0001.

The CD56^dim^ NK cell population revealed a significant increase of PD-1 expression in treatment-naive patients compared to HD (HD *vs.* CC pre-treatment: *p* = 0.0457), which was further amplified post-treatment (HD *vs.* CC post-treatment: *p* < 0.0001; CC pre-treatment *vs.* CC post-treatment: *p* = 0.0261). A similar trend was noted for LAG-3 (HD *vs.* CC pre-treatment: *p* = 0.0313; HD *vs.* CC post-treatment: *p* < 0.0001; CC pre-treatment *vs.* CC post-treatment: *p* = 0.0306) and BTLA (HD *vs.* CC pre-treatment: *p* = 0.0051; HD *vs.* CC post-treatment: *p* < 0.0007). However, no significant difference was observed in BTLA expression between pre- and post-treatment patient groups (CC pre-treatment *vs.* CC post-treatment: *p* = 0.4516). No statistically significant differences were found for the markers TIM-3, TIGIT, and NKG2A between the groups (**Figure 6A**).

In the CD56^bright^ NK cell population, a similar pattern of immune checkpoint expression was observed, with an increase in the percentage of positive cells in the CC pre-treatment group, followed by an exacerbation post-treatment, compared to the HD group. However, these differences did not reach statistical significance between the two CC patient subgroups. Notably, this trend was particularly evident for LAG-3 (HD *vs.* CC pre-treatment: *p* < 0.0001; HD *vs.* CC post-treatment: *p* < 0.0001; CC pre-treatment *vs.* CC post-treatment: *p* = 0.5857) and TIM-3 (HD *vs.* CC pre-treatment: *p* = 0.0018; HD *vs.* CC post-treatment: *p* = 0.0008; CC pre-treatment *vs.* CC post-treatment: *p* = 0.6651). Though TIGIT followed a similar trend, statistical significance was only reached in the post-treatment group compared to HD (HD *vs.* CC pre-treatment: *p* = 0.1038; HD *vs.* CC post-treatment: *p* = 0.0148; CC pre-treatment *vs.* CC post-treatment: *p* = 0.3632). Interestingly, PD-1 expression increased significantly in both CC groups compared to HD. Post-treatment patients exhibited a decrease in PD-1 expression compared to pre-treatment, although this did not reach statistical significance (HD *vs.* CC pre-treatment: *p* < 0.0001; HD *vs.* CC post-treatment: *p* = 0.0002; CC pre-treatment *vs.* CC post-treatment: *p* = 0.2414). No significant changes were observed in the percentage of cells positive for NKG2A and BTLA between the groups (**Figure 6B**).

**Figure 7:**
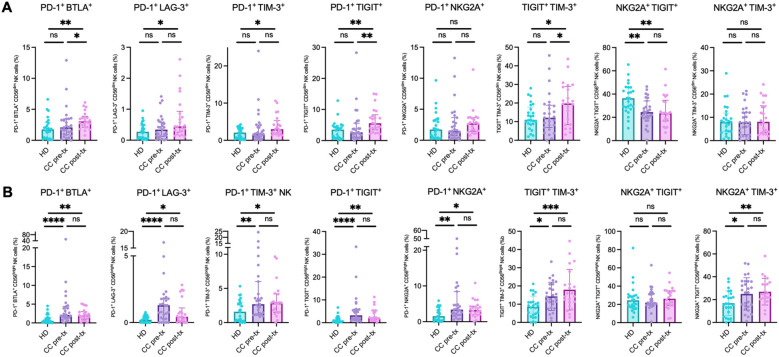
Co-expression of inhibitory immune checkpoint markers in NK cells from peripheral blood of healthy donors (HD), CC patients before treatment (CC pre-tx), and CC patients after treatment (CC post-tx). **(A)** Percentage of peripheral CD56^dim^ NK cells expressing co-inhibitory immune checkpoints. **(B)** Percentage of peripheral CD56^bright^ NK cells expressing co-inhibitory immune checkpoints: PD-1^+^BTLA^+^, PD-1^+^LAG-3^+^, PD-1^+^TIM-3^+^, PD-1^+^TIGIT^+^, TIGIT^+^TIM-3^+^, NKG2A^+^TIGIT^+^, PD-1^+^NKG2A^+^, and NKG2A^+^TIM-3^+^. Comparisons between groups were performed using one-way ANOVA for parametric variables: NKG2A^+^TIM-3^+^ CD56^dim^ and CD56^bright^ and TIGIT^+^TIM-3^+^ CD56^bright^, and Kruskal-Wallis for all other analyzed co-expressions, followed by Benjamini-Hochberg FDR correction for multiple comparisons. Data is shown as individual percentages of cells positive for co-expression with mean ± SD for parametric variables and median with IQR for non-parametric variables. *p ≤ 0.05, **p≤ 0.01, ***p ≤0.001, ****p ≤0.0001.

The co-expression analysis revealed consistent evidence of a putative exhausted phenotype in NK cells across both patient groups, with a notable exacerbation following treatment, especially in the CD56^dim^ population. Significant increases were detected in the co-expression of several inhibitory receptors: PD-1^+^BTLA^+^ (HD *vs.* CC pre-treatment: *p* = 0.3513; HD vs. CC post-treatment: *p* = 0.0029; CC pre-treatment *vs.* CC post-treatment: *p* = 0.0329), PD-1^+^LAG-3^+^ (HD *vs.* CC pre-treatment: *p* = 0.3683; HD vs. CC post-treatment: *p* = 0.0105; CC pre-treatment *vs.* CC post-treatment: *p* = 0.0829), PD-1^+^TIM-3^+^ (HD *vs.* CC pre-treatment: *p* = 0.3798; HD *vs.* CC post-treatment: *p* = 0.0063; CC pre-treatment *vs.* CC post-treatment: *p* = 0.0533), PD-1^+^TIGIT^+^ (HD vs. CC pre-treatment: *p* = 0.7292; HD *vs.* CC post-treatment: *p* = 0.0025; CC pre-treatment *vs.* CC post-treatment: *p* = 0.0061), and TIGIT^+^TIM-3^+^ (HD *vs.* CC pre-treatment: *p* = 0.9917; HD *vs.* CC post-treatment: *p* = 0.0488; CC pre-treatment vs. CC post-treatment: *p* = 0.0438). In contrast, the co-expression of NKG2A^+^TIGIT^+^ showed a different trend, with a significant decrease in both patient groups compared to healthy controls (HD *vs.* CC pre-treatment: *p* = 0.0017; HD *vs.* CC post-treatment: *p* = 0.0054; CC pre-treatment *vs.* CC post-treatment: *p* = 0.8195). No significant differences were found in the co-expressions of PD-1^+^NKG2A^+^ and NKG2A^+^TIM-3^+^ (**Figure 7A**).

In CD56^bright^ NK cells, a significant increase in populations exhibiting signs of immune exhaustion was observed, including PD-1^+^BTLA^+^ (HD *vs.* CC pre-treatment: *p* = 0.0001; HD *vs.* CC post-treatment: *p* = 0.0048), PD-1^+^LAG-3^+^ (HD *vs.* CC pre-treatment: *p* < 0.0001; HD *vs.* CC post-treatment: *p* = 0.0191), PD-1^+^TIM-3^+^ (HD *vs.* CC pre-treatment: *p* = 0.0064; HD *vs.* CC post-treatment: *p* = 0.0261), PD-1^+^TIGIT^+^ (HD *vs.* CC pre-treatment: *p* = 0.0001; HD *vs.* CC post-treatment: *p* = 0.0052), PD-1^+^NKG2A^+^ (HD *vs.* CC pre-treatment: *p* = 0.0013; HD *vs.* CC post-treatment: *p* = 0.0108), TIGIT^+^TIM-3^+^ (HD *vs.* CC pre-treatment: *p* = 0.0170; HD *vs*. CC post-treatment: *p* = 0.0003), and NKG2A^+^TIM-3^+^ (HD *vs.* CC pre-treatment: *p* = 0.0216; HD *vs.* CC post-treatment: *p* = 0.0073), compared to the healthy control group. No significant differences were found between the pre- and post-treatment patient subgroups. Consistent with the individual PD-1 expression patterns observed in this cell population, post-treatment patients tended to decrease the percentage of PD-1^+^ cells co-expressing other immune checkpoints compared to pre-treatment patients. Nevertheless, these changes did not reach statistical significance (**Figure 7B**).

### Dysbiosis score positively correlates with NK cell exhaustion scores

To evaluate the association between microbiota dysbiosis and NK cell exhaustion, a Spearman correlation analysis was performed between the dysbiosis score and NK cell exhaustion scores derived from CD56^dim^ and CD56^bright^ subpopulations, as well as their global exhaustion score. A positive correlation was observed between the dysbiosis score and the global NK cell exhaustion score (R = 0.50, *p* < 0.0001), suggesting that higher levels of dysbiosis are associated with increased NK cell exhaustion when using this approach. When analyzing individual NK cell subsets, the dysbiosis score showed a significant positive correlation with both CD56^dim^ (R = 0.44, *p* < 0.0001) and CD56^bright^ NK cell exhaustion scores (R = 0.42, *p* < 0.00), indicating that alterations in microbiota composition could be linked to exhaustion across different NK cell populations (**Supplementary Figure 5**).

### Prediction of newly diagnosed cervical cancer patients with a machine-learning approach

**Figure 8:**
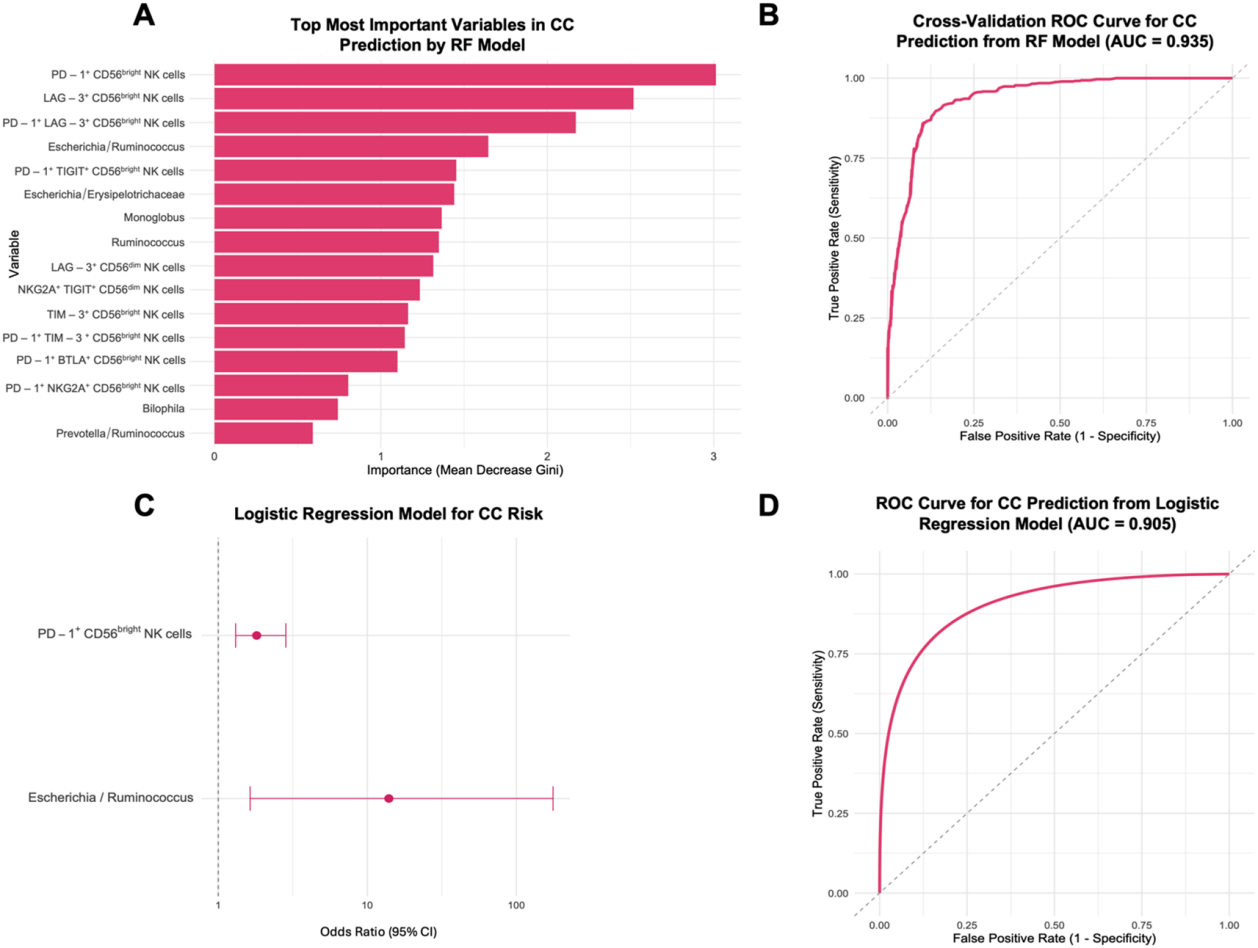
Prediction of CC risk using a Random Forest model and logistic regression from healthy donors (HD), and CC patients before treatment (CC pre-tx). **(A)** Top 16 most important variables from 104 tested for CC prediction: The most important variables in predicting CC risk are displayed according to the RF model’s Mean Decrease Gini scores. **(B)** ROC Curve for CC prediction from RF. **(C)** Forest Plot for factors associated with CC risk: OR and 95% CI from a logistic regression model using PD-1^+^ CD56^bright^ NK cells and *Escherichia*/*Ruminococcus* ratio for CC risk. **(D)** ROC Curve for CC prediction from logistic regression model. RF, Random Forest; ROC, Receiver Operating Characteristic; CI, confidence intervals; OR, Odds ratio.

The random forest (RF) classification model identified the most important predictors for CC as the expression of PD-1, LAG-3, and their co-expression on CD56^bright^ NK cells, along with the *Escherichia*/*Ruminococcus* ratio (**Figure 8A**). The model demonstrated robust discriminative power with an average ROC-AUC of 0.950 (SD = 0.0545). Repeated 10-fold cross-validation (5 repetitions) confirmed the stability and reliability of the model, with a mean ROC-AUC of 0.935 (**Figure 8B**). This RF model effectively distinguished pre-treatment CC patients from HD, as shown by the confusion matrix (**Supplementary Figure 6**), achieving an accuracy of 92.86% (*p* = 0.00455, 95% CI: 0.6613-0.9982), with a kappa coefficient of 0.8571. The model yielded a sensitivity of 87.5%, specificity of 100%, positive predictive value of 100%, and negative predictive value of 85.71%, resulting in a balanced accuracy of 93.75%. Individual ROC curve analyses were performed for the five top-ranked variables identified by the RF model. These features exhibited moderate-to-high discriminative power, with AUCs ranging from 0.778 to 0.891 (**Supplementary Figure 7**).

Subsequent simplified logistic regression using the top predictors stepwise selected revealed that increased PD-1 expression on CD56^bright^ NK cells (OR = 1.81, 95% CI: 1.31-2.84, *p* = 0.002) and a higher *Escherichia*/*Ruminococcus* ratio (OR = 14.0, 95% CI: 1.64-176, *p* = 0.023) were significantly associated with increased CC risk (**Figure 8C**). This logistic regression model yielded an AUC of 0.905 (**Figure 8D**). The final predictive equation was:


$$logit\left(P\right)=-3.3919+0.5938\;\times \;PD-1^{+}CD56^{bright}\;NK\;cells+2.6358\times \frac{Escherichia}{Ruminococcus}ratio\;\;$$

### Predictive modeling of mortality in newly diagnosed cervical cancer patients

**Figure 9:**
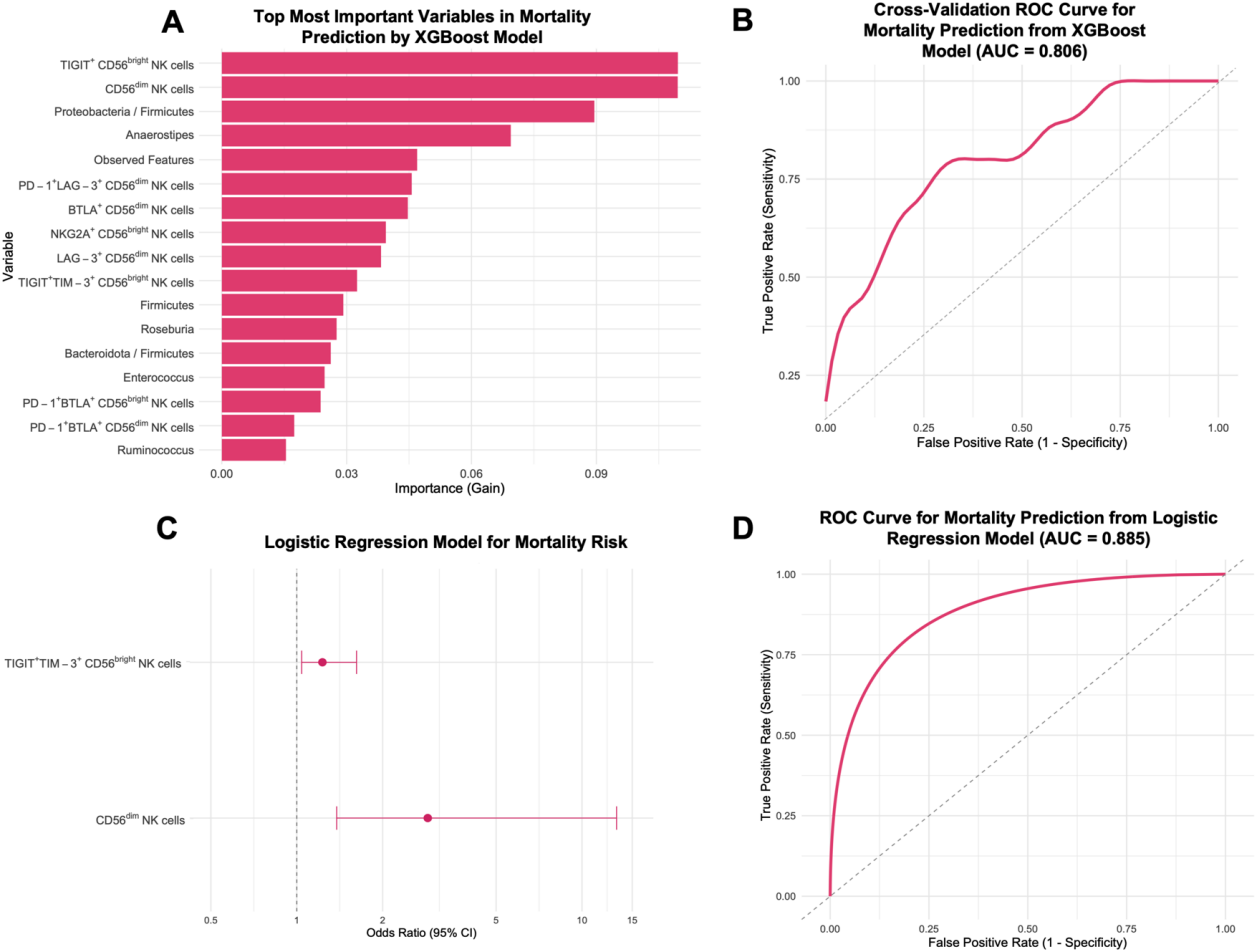
Prediction of mortality risk in CC patients before treatment (CC pre-tx) using XGBoost model and logistic regression. **(A)** Top 17 most important variables from 106 tested for CC mortality prediction: The most important variables in predicting mortality risk are displayed according to the XGBoost importance. **(B)** ROC Curve for CC mortality prediction from XGBoost. **(C)** Forest Plot for factors associated with CC mortality risk: OR and 95% CI from a logistic regression model using TIGIT^+^TIM-3^+^ CD56^bright^ NK cells and CD56^dim^ NK cells for mortality risk. **(D)** ROC Curve for CC mortality prediction from the logistic regression model. XGBoost, eXtreme Gradient Boosting; ROC, Receiver Operating Characteristic; CI, confidence intervals; OR, Odds ratio.

An XGBoost classification model was applied to predict survival outcomes in CC patients. Feature importance analysis identified the most relevant predictors as the expression of TIGIT^+^ CD56^bright^ NK cells, CD56^dim^ NK cells, and the *Proteobacteria*/*Firmicutes* ratio, followed by other immune and microbial features (**Figure 9A**). The model achieved an AUC of 0.875 (SD = 0.236). Repeated cross-validation yielded a mean ROC-AUC of 0.806 (SD = 0.119) (**Figure 9B**). Despite a limited sample size, this model effectively distinguished survival from death in CC patients, as shown by the confusion matrix (**Supplementary Figure 8**). The model achieved an accuracy of 83.3% (95% CI: 0.3588-0.9958), with a sensitivity of 75.0%, specificity of 100%, balanced accuracy of 87.5%, and a kappa value of 0.6667.

The stepwise algorithm revealed TIGIT^+^TIM-3^+^ CD56^bright^ and CD56^dim^ NK cell frequency as the most important variables. Posterior logistic regression showed that elevated TIGIT^+^TIM-3^+^ CD56^bright^ (OR = 1.23, 95% CI: 1.04-1.62, *p* = 0.050) and CD56^dim^ NK cells (OR = 2.89, 95% CI: 1.38-13.22, *p* = 0.0499) were associated with higher mortality (**Figure 9C**). The two-variable model yielded an AUC of 0.885 (**Figure 9D**). The final predictive equation was:


$$logit\left(P\right)=-105.640+0.208\times \;TIGIT^{+}TIM-3^{+}\;CD56^{bright}\;NKcells+1.058\times \;CD56^{dim}NK\;cells\;\;$$

A second logistic regression using TIGIT^+^TIM-3^+^ CD56^bright^ NK cells and *Proteobacteria*/*Firmicutes* (log) ratio did not reveal any statistically significant associations with mortality (**Supplementary Figure 9A**). The combined model yielded an AUC of 0.823 (**Supplementary Figure 9B**), but these findings should be considered preliminary.

### Survival analysis in newly diagnosed cervical cancer patients

Kaplan–Meier survival analysis was conducted over a 15-month follow-up period to evaluate the prognostic significance of the identified markers. Analyses were restricted to pre-treatment CC patients, as RCT profoundly alters both the gut microbiota and NK cell phenotypes; therefore, untreated samples provide the most appropriate setting to assess predictors of survival. Of the 27 pre-treatment CC patients, 23 were evaluable for survival analysis. 4 patients were lost to follow-up and, therefore, excluded from survival analyses. Patients alive at the end of the 15-month follow-up were censored at that time. Patients were stratified by cut-off values determined via ROC curve analysis for each variable. Patients were stratified into high and low groups using cut-off values derived from ROC curves based on the survival status of pre-treatment CC patients, selecting the point with maximum sensitivity and specificity for each variable.

**Figure 10:**
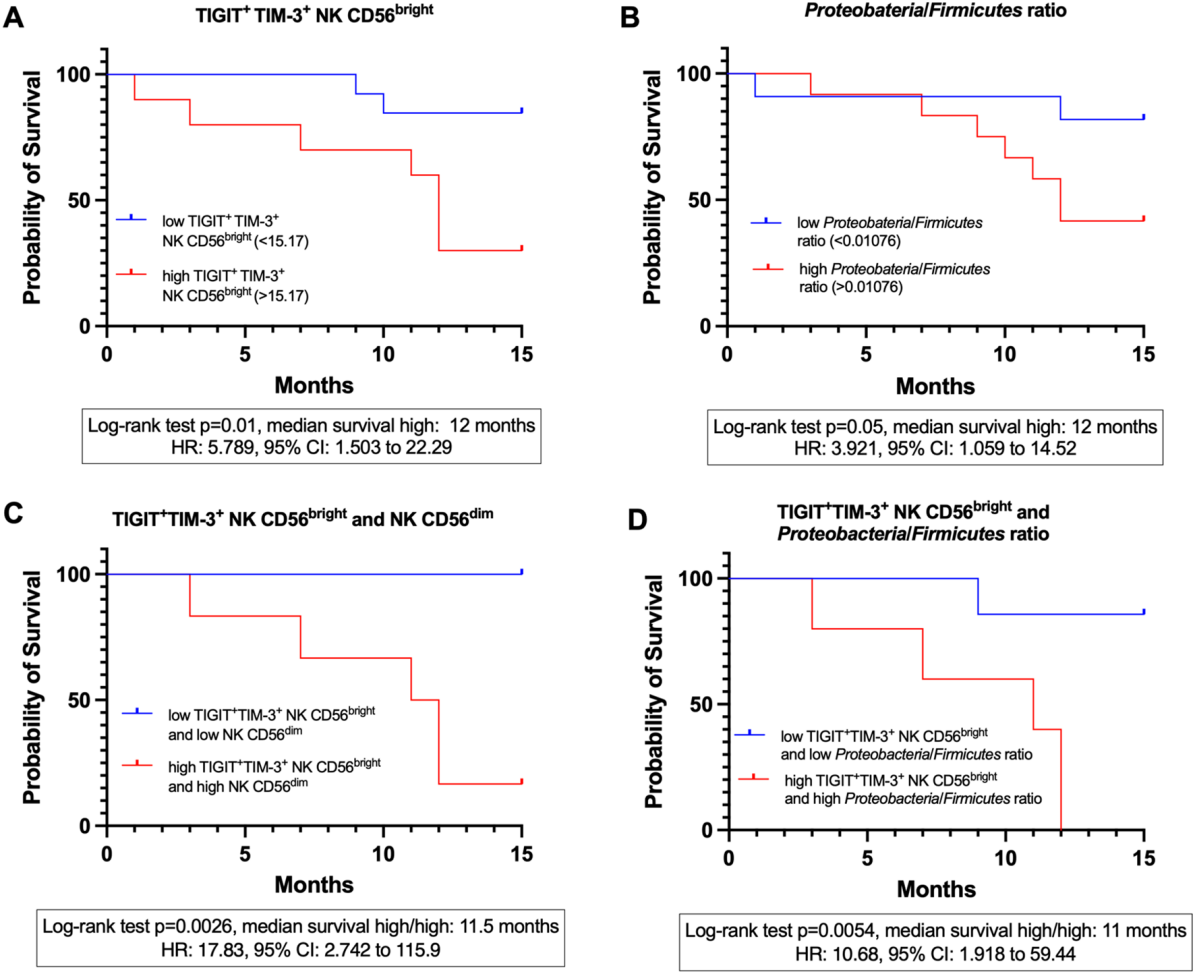
Kaplan-meier survival curves at 15 months in CC patients before treatment (CC pre-tx). **(A)** 15-month survival stratified by the expression of TIGIT^+^TIM-3^+^ on CD56^bright^ NK cells. **(B)** 15-month survival stratified by the *Proteobacteria*/*Firmicutes* ratio. **(C)** 15-month survival stratified by the combined expression of TIGIT^+^TIM-3^+^ on CD56^bright^ NK cells and the percentage of CD56^dim^ NK cells. **(D)** 15-month survival stratified by the combined expression of TIGIT^+^TIM-3^+^ on CD56^bright^ NK cells and the *Proteobacteria*/*Firmicutes* ratio.

Patients with elevated levels of TIGIT^+^TIM-3^+^ expression on CD56^bright^ NK cells were associated with worse prognosis, with a median survival of 12 months compared to higher survival in the low-expression group (log-rank p=0.01), and a log-rank hazard ratio (HR) of 5.789 (95% CI: 1.503-22.29). In this analysis, 7 deaths occurred in the high-expression group and 2 in the low-expression group, while 3 and 11 patients, respectively, were censored at 15 months (**Figure 10A**). Similarly, a high *Proteobacteria*/*Firmicutes* ratio exhibited significantly reduced overall survival (log-rank *p* = 0.05), with a median survival of 12 months and a log-rank HR of 3.921 (95% CI: 1.059-14.52). For this variable, 7 deaths occurred in the high-ratio group and 2 in the low-ratio group, while 5 and 9 patients, respectively, were censored at 15 months (**Figure 10B**). An analysis for the CD56^dim^ NK cell population was performed; patients with a high percentage showed a trend toward lower survival probability (log-rank HR = 2.436), but the difference was not statistically significant (log-rank *p* = 0.1831; 95% CI: 0.6553-9.055) (not shown).

### Combined marker analyses

Combined survival analyses were conducted to assess synergistic effects; as these analyses were restricted to extreme categories (high/high vs. low/low variables), the effective sample size was reduced. Patients with high levels of both TIGIT^+^TIM-3^+^ expression on CD56^bright^ NK cells and a high frequency of CD56^dim^ NK cells had a significantly reduced survival with a mean of 11.5 months (Mantel-Haenszel HR = 17.83, 95% CI: 2.742-115.9, log-rank *p* = 0.0026) compared to those with low levels of both variables. In this comparison, 5 deaths occurred in the high/high group, whereas no deaths were observed in the low/low group; 7 patients in the low/low group and 1 in the high/high group were censored at 15 months (**Figure 10C**). Similarly, patients presenting both high TIGIT^+^TIM-3^+^ expression on CD56^bright^ NK cells and a high *Proteobacteria*/*Firmicutes* ratio exhibited significantly reduced survival, with a mean of 11 months (HR = 10.68, 95% CI: 1.918-59.44, log-rank *p* = 0.0054), compared to patients with low values for both markers. For this variable combination, 5 deaths occurred in the high/high group and 1 in the low/low group, while 6 patients in the low/low group and none in the high/high group were censored at 15 months (**Figure 10D**).

## Discussion

This study provides novel evidence that gut microbial imbalances and peripheral NK cell immune exhaustion play a central role in cervical cancer (CC) progression. Furthermore, the integrative analysis of these two variables supports the diagnosis and prognosis of CC. By integrating microbiota profiling, immunophenotyping, and computational analyses, we identified a dysbiotic microbial signature and a putative NK cell exhausted phenotype that radio-chemotherapy (RCT) further exacerbates.

Congruent with previous reports, the expansion of *Proteobacteria, Prevotella*, *Escherichia-Shigella*, *Streptococcus*, and *Enterococcus* has been observed in the gut microbiota of CC patients (7, 8). In our study, CC patients harbored a gut microbiota with significantly reduced richness, diversity, and evenness, accompanied by an increased dominance of pro-inflammatory pathobionts such as *Prevotella* and *Escherichia-Shigella*. These genera have been associated with inflammatory diseases, immune evasion, cancer progression, and therapy resistance (49–51). Notably, *Enterococcus* and *Bilophila* were enriched in pre-treatment CC patients. *Enterococcus faecalis* has been shown to polarize colonic macrophages toward a clastogenic M1 phenotype, promoting DNA damage via bystander effect (52), while *Bilophila wadsworthia* has been associated with genotoxicity and cancer development through the production of hydrogen sulfide, a microbial metabolite capable of inducing epithelial damage, DNA instability, and protumorigenic signaling (53). Although many of these taxa are considered commensal, under dysbiotic conditions, they can express a range of virulence factors, including adhesins, hemolysins, LPS, and proteases, that contribute to inflammation and immune modulation (54).

Conversely, health-associated and SCFA-producing taxa such as *Ruminococcus*, *Christensenellaceae*, and *Eubacterium* were depleted; the reduction of these bacteria has been linked with disease (55). SCFAs such as acetate, propionate, and butyrate play a central role in maintaining mucosal integrity and modulating immune responses (20). These metabolites can enhance NK proliferation, cytotoxicity, and extracellular vesicle secretion (56), and boost CD8^+^ T cell effector responses, increasing TNF-α and IFN-γ secretion (57). Although certain contexts suggest SCFAs may also dampen immune activation by upregulating inhibitory markers (58), in the setting of CC, the observed loss of SCFA-producing bacteria likely reflects an immunologically impaired environment that favors inflammation and tumor evasion. However, functional assays will be essential to establish whether SCFAs differentially influence NK cell subsets and shape their exhaustion and effector potential.

Furthermore, RCT intensified this dysbiosis by depleting even more health-associated taxa such as *Ruminococcus* and *Clostridium*, while enriching species like *Phascolarctobacterium*, which has been previously associated with oncogenic processes (59).

Functional prediction of microbial metabolism revealed a proinflammatory and stress-associated profile in CC patients, with enrichment of pathways related to LPS biosynthesis, enterobactin production, and stress response pathways. Elevated enterobactin biosynthesis, a siderophore produced by *Escherichia*, may reflect adaptation to iron-rich environments, potentially favored by CC related bleeding (60). The enrichment of pathways associated with bacterial strategies for coping with nutrient limitation and stress, such as glyoxylate bypass, chorismate pathway and biosynthesis of ppGpp alarmone suggests a metabolically constrained environment in CC patients, where microbes reduce anabolic processes, activating stringent response and processes such as rRNA synthesis and shift toward carbon conservation pathways, promoting the growth of bacteria couped to these processes like *Escherichia* (61–63). The enrichment of histidine degradation pathways may be relevant, as its metabolite imidazole propionate has been implicated in promoting intestinal inflammation by activating pro-inflammatory signaling pathways, increasing nitric oxide synthesis and IL-6 levels, and impairing mucosal integrity by reducing goblet cell populations (64). Upregulation of toluene degradation pathways suggests microbial adaptation to xenobiotic-rich environments, potentially leading to the production of toxic metabolites like cresols that may disrupt gut homeostasis and contribute to carcinogenic processes (65, 66).

Chorismate metabolism, enriched in CC patients, encompasses the microbial biosynthesis of enterobactin, ubiquinol, and L-tryptophan (67). L-tryptophan biosynthesis, also upregulated in CC samples, can be further catabolized by indoleamine 2, 3-dioxygenase (IDO) and tryptophan 2, 3-dioxygenase (TDO), which are homologous enzymes. While IDO is mainly expressed by mammalian cells, TDO is widely distributed across both eukaryotes and prokaryotes, including certain bacterial taxa (68). These enzymes generate immunoregulatory metabolites such as kynurenine, picolinic acid, and quinolinic acid, which inhibit the proliferation and cytotoxic activity of activated NK cells (69). Notably, bacterial degradation of L-tryptophan to kynurenine occurs under aerobic conditions (70), suggesting a relationship with the intestinal dysbiosis observed in CC patients, since we have observed a greater proliferation of facultative aerobic genera. Moreover, commensal-derived butyrate has been shown to downregulate IDO-1 expression in intestinal epithelial cells (71). Congruently, we observed a depletion of butyrate-producing bacteria in CC patients, which may favor an intestinal microenvironment permissive to IDO overexpression. Kynurenine, in turn, can enter NK cells through the aryl hydrocarbon receptor (AhR) and downregulate activating receptors such as NKG2D and NKp46 through STAT1/3 signaling, ultimately impairing NK cell function (72). Additionally, chorismate is also a precursor for the bacterial biosynthesis of immunosuppressants such as FK506, FK520, and rapamycin, further supporting its role in modulating host immune responses (73).

Interestingly, we observed overexpression of the L-arginine/L-ornithine degradation pathway in pre-treatment samples. Arginase is a key enzyme in the urea cycle that transforms L-arginine into urea and L-ornithine. Two isoforms of arginase, ARG1 and ARG2, are aberrantly expressed in various types of cancer and have been shown to play a crucial role in regulating tumor growth and metastasis (74). Additionally, L-arginine depletion has been reported to impair several critical functions of NK cells, including their proliferation, cytotoxic activity, IFN-γ production, and the expression of NKp46 and NKp30 (75). Recent findings have shown that *Proteobacteria* can directly consume arginine, thereby reducing its systemic availability and impairing anti-tumor immunity by enhancing Treg suppressive activity and dampening CD8^+^ T cell responses (76). Treg cells are also known to inhibit NK cell functions (77). Consistent with these reports, our data revealed an increased abundance of both the phylum *Proteobacteria* and the genus *Escherichia*, a representative member known to degrade arginine under nitrogen-limiting conditions (78). Notably, the enrichment of this pathway co-occurred with increased allantoin degradation and ppGpp biosynthesis, both of which are associated with bacterial adaptation to nitrogen scarcity (79, 80). These findings suggest that alterations in L-arginine metabolism may contribute to tumor progression and immune evasion by compromising not only CD8^+^ T cells but also NK cell-mediated responses. Further investigation should aim to address L-arginine metabolism in CC and its impact on the tumor microenvironment (TME).

Allantoin degradation was also upregulated in CC patients, particularly post-treatment. Allantoin is produced via non-enzymatic conversion of uric acid mediated by reactive oxygen species and is considered a biomarker of systemic oxidative stress in humans (81). Elevated oxidative status has been clinically documented in CC patients undergoing RCT (82). Under nitrogen-limiting conditions, certain bacteria degrade allantoin as an alternative nitrogen source (80). The upregulation of the allantoin degradation pathway by the gut microbiota may reflect microbial adaptation to this oxidative environment. Moreover, allantoin has been shown to impair cisplatin efficacy via direct interaction (83), suggesting that this microbial mechanism could potentially enhance treatment response. However, allantoin also exerts antioxidant and mucosal-protective effects (84), and its excessive degradation may compromise epithelial homeostasis, potentially contributing to post-RCT mucosal damage. Finally, the post-treatment enrichment of antioxidant biosynthesis pathways, such as ubiquinol production, may represent microbial adaptations to the oxidative and inflammatory conditions induced by RCT (85). These findings highlight the functional plasticity and resilience of the microbiota in response to treatment-related stress.

In parallel, we observed profound alterations of peripheral NK cell phenotypes, particularly following treatment. Both CD56^dim^ and CD56^bright^ NK cell subsets exhibited increased expression and co-expression of inhibitory checkpoint receptors, including PD-1, LAG-3, TIM-3, TIGIT, and BTLA. This phenotype was especially pronounced after RCT, particularly in the CD56^dim^ population. Notably, co-expression patterns were elevated in CD56^dim^ NK cells post-treatment, while CD56^bright^ NK cells showed a reduction, yet remained significantly higher than in HD. These findings highlight the potential benefit of ICB therapies in CC, particularly targeting PD-1 and other inhibitory axes, as suggested by clinical data supporting the efficacy of anti-PD-1 agents in this setting (86). NK cells go far beyond the classical dichotomy of cytotoxic CD56^dim^ and regulatory CD56^bright^ (87). Additional subsets such as CD56^bright^CD16^+^, CD56^dim^CD16^-^, CD56^-^CD16^+^, and CD56^superbright^ populations illustrate their remarkable plasticity. For example, CD56^bright^ cells can acquire cytotoxic activity after IL-15 priming, hepatic CD56^bright^ NK cells show reduced cytokine secretion despite their phenotype, uterine CD56^superbright^ cells promote angiogenesis and tissue remodeling (88), and CD56^-^CD16^+^ have been shown to expand in cancer (89). Together, these observations highlight that surface phenotype alone does not define NK cell functional fate, which is increasingly understood in the context of maturation, metabolism, and high-dimensional subset frameworks (90, 91).

The concept of NK cell exhaustion in cancer has gained considerable attention in recent years, particularly given its correlation with poor prognosis and relevance to ICB responsiveness. Although the definition of exhaustion in NK cells is not as well established as in T cells, hallmark features include downregulation of activating receptors, impaired proliferation and cytokine production, and sustained upregulation of inhibitory molecules (92). Accordingly, our findings of upregulated PD-1, LAG-3, BTLA, TIM-3, and TIGIT, particularly in co-expression patterns, support a putatively exhausted state.

Following treatment, the immune landscape undergoes further alteration. Radiation therapy (RT) has been shown to increase PD-L1 expression on cancer cells (93) and to modulate immune checkpoint profiles in both tumor and peripheral immune cells. For example, PD-1 and LAG-3 expression increased in T cells of rectal cancer patients after RT (94). Other tumor evasion mechanisms include shielding via platelets or collagen, and upregulation of ligands such as CD155, which binds TIGIT on NK cells, impairing their function (95). Additionally, microbial influences on the tumor microenvironment are emerging. Intratumoral bacteria like *Fusobacterium nucleatum*, a bacterium linked to colorectal cancer, can suppress anti-tumor immunity by binding to TIGIT via its Fap-2 protein, thereby blocking tumor cell elimination (96).

To explore the interplay between microbial alterations and NK cell phenotypes, we developed composite dysbiosis and NK exhaustion scores. These scores revealed a significant positive correlation, suggesting that microbial dysbiosis may contribute to or exacerbate NK cell exhaustion. While causality remains to be established, several mechanistic routes may underlie this link. NK cells are equipped with pattern recognition receptors and can directly respond to microbial components such as LPS, flagellin, and outer membrane proteins via non-TLR4 pathways, TLR5, TLR2, respectively, often in synergy with cytokines like IL-1β, IL-2, IL-12, and IL-15 (97, 98). Additionally, direct interactions between microbial ligands and natural cytotoxicity receptors on NK cells have been documented. Specifically, NKp44 has been shown to directly bind to *Mycobacterium*, *Nocardia farcinica*, and *Pseudomonas aeruginosa* (99), NKp46 to *Fusobacterium nucleatum* (100), and NKp30 to β-1, 3-glucans on *Candida albicans* and *Cryptococcus neoformans* (101).

Dysbiosis-induced intestinal permeability may facilitate the translocation of microbial antigens into circulation, driving chronic NK cell activation and potential exhaustion (102, 103). In murine models of colorectal cancer, dysbiosis has been associated with T cell exhaustion, characterized by an increased population of PD-1^+^LAG-3^+^TIM-3^+^ CD8^+^ T cells, suggesting that microbial imbalance may promote tumor progression through immune dysfunction (21). Our data revealed enrichment of LPS synthesis pathways in pre-treatment patients. LPS can disrupt intestinal barriers by activating myosin light chain kinase and enhancing paracellular translocation (104). Consistently, our data reveals an increase in the *Proteobacteria* phylum in CC, which are recognized as major producers of LPS.

NK cell activation depends not only on microbial ligands but also on the priming by accessory cells, including dendritic cells (DCs), monocytes, and macrophages, as well as on a cytokine milieu composed of IL-12, IL-15, type I IFNs, and IL-18 (105). Commensal microbiota is crucial in shaping this axis. Ganal et al. demonstrated that mononuclear phagocyte-mediated NK cell priming via IFN-I is microbiota-dependent. Germ-free mice failed to induce NK cell cytotoxicity due to a lack of microbiota-induced chromatin remodeling in DCs, which impairs the accessibility of transcription factors such as IRF3 and NF-κB to IFN-I promoter regions. Failing to produce IFN-I, a cytokine essential for NK cell priming by subsequent IL-15 trans-presentation. Importantly, this defect was reversible upon microbiota colonization (106). Moreover, microbiota-derived cyclic di-AMP can stimulate STING-mediated IFN-I production in monocytic cells, recruiting DCs and promoting IL-15-mediated NK cell activation within the tumor microenvironment (107).

The ratio of *Escherichia*/*Ruminococcus*, together with the elevated PD-1^+^ CD56^bright^ NK cells, emerged as the strongest predictors of CC in our RF and logistic regression models. Notably, an increased abundance of *Escherichia* and a concomitant reduction in *Ruminococcus* have also been reported in previous CC microbiota studies, supporting the consistency of this dysbiotic pattern across independent cohorts (10). As already mentioned, *Escherichia*, a facultative aerobic pathobiont, is associated with inflammation and genotoxin production (50), while *Ruminococcus* contributes to gut homeostasis through SCFA production and epithelial maintenance (108). In parallel, elevated PD-1 expression on CD56^bright^ NK cells may indicate an impairment of cytokine secretion and, consequently, a reduction in the infiltration of other immune cells, such as DCs, into the TME, leading to compromised immune surveillance (107). This microbial-immune axis underscores the importance of integrating microbiota composition and immune phenotypes for possible disease classification through potential non-invasive biomarkers for CC risk.

Notably, similar microbiota-NK interactions have been reported in other malignancies. In melanoma, the combination of TIGIT^+^ NK cells with specific gut microbial profiles predicted response to checkpoint blockade, directly linking microbiota composition with NK-mediated immunotherapy outcomes (47). In prostate cancer, microbiota-informed NK cell biomarkers, such as upregulation of PD-1, TIM-3, increased CD56^bright^, and downregulated NKG2D, have been proposed to refine prognosis and guide clinical management strategies (109). In hepatocellular carcinoma, modulation of the gastrointestinal microbiota enhanced NK cell activity and reduced exhaustion (110). These studies highlight microbiota-driven regulation of NK function as a broader phenomenon across cancers, with our work providing the first integrative evidence in CC.

Altogether, these findings reveal a dysbiotic gut microbiota profile in CC patients, marked by a reduction of diversity and expansion of pro-inflammatory taxa, which is further aggravated following RCT. This microbial imbalance may promote systemic inflammation and immune dysregulation, potentially influencing the vaginal ecosystem and facilitating persistent HPV infection. These results underscore the importance of the gut-immune-vaginal axis in CC pathogenesis and point toward the potential of microbiota modulation as a complementary approach in disease management. This study offers an integrative view of microbial and immunological alterations in CC and provides a rationale for future translational efforts targeting the microbiota-immune axis. Given the pivotal role of gut microbiota in shaping immune responses and influencing therapeutic efficacy, as well as the fundamental role of NK cells in anti-tumor immunity, understanding how dysbiosis affects NK cell function may unlock new microbiota-focused strategies to enhance treatment outcomes.

The integration of microbiota and NK cell profiling holds great potential for precision medicine in CC. Beyond characterizing the established disease, these approaches could be extended to women with persistent HPV infection and precancerous lesions, to determine whether microbial-immune alterations precede malignant transformation. In parallel, composite indices such as the *Escherichia*/*Ruminococcus* ratio or NK exhaustion scores require validation as predictive biomarkers across larger and longitudinal cohorts. This aligns with previous approaches such as the Royal Marsden Hospital (RMH) Score, which integrates albumin, lactate dehydrogenase (LDH) levels, and the number of metastatic sites, and has been validated as a prognostic tool in oncology (111), as well as systemic indicators of nutritional and inflammatory status (112, 113) which may also interact with microbial profiles in shaping clinical outcomes.

Importantly, strategies to restore microbial balance through probiotics, prebiotics, or diet may represent feasible interventions to modulate NK cell function and improve therapeutic responses. In the next years, advancing from correlative studies to interventional designs will be crucial to translate the microbiota-immune axis into an actionable tool for patient stratification and treatment optimization. Overall, this study supports the rationale for microbiota-targeted interventions as adjunctive strategies in CC, although prospective validation is required.

The limitations of this study include a relatively small sample size and the use of a cross-sectional rather than a longitudinal design, which restricts the ability to assess temporal dynamics and infer causal relationships. Moreover, incorporating clinical and lifestyle data, such as antibiotic use and dietary habits, will be essential for a more comprehensive understanding of microbiota-host interactions in CC. Although this study focused on the potential influence of the gut microbiota on systemic NK cell exhaustion, it did not include analysis of vaginal or intratumoral microbiota. This limits the ability to directly assess microbial translocation or characterize local dysbiosis within the tumor environment. Future studies should incorporate paired analyses of gut, vaginal, and intratumoral microbiota, along with immune profiling within the TME, including NK cell exhaustion and other immunological parameters, to establish more precise associations. Another important limitation relates to the immunophenotypic characterization of NK cells. In this study, immune checkpoint expression was analyzed in the two NK cell subpopulations CD56^dim^ and CD56^bright^, a strategy that only partially captures their currently recognized heterogeneity. A more refined approach should distinguish functional NK subsets and incorporate a broader panel of activating and inhibitory receptors to better characterize exhaustion phenotypes. In addition, the global NK exhaustion and dysbiosis scores used here represent an exploratory composite that requires validation in larger cohorts and diverse contexts. Complementary functional assays, including degranulation, cytokine production, and cytotoxicity measurements, will be essential to extend these findings, particularly when applied to distinct NK subsets that may differentially respond to microbiota alterations, consequently shaping their exhaustion and effector capacity.

## Data Availability

The original contributions presented in the study are publicly available. This data can be found in NCBI: https://www.ncbi.nlm.nih.gov/bioproject/PRJNA1347852.
