# Supplementary material for: Microbiota–immune dysregulation in cervical cancer patients from Western Mexico: linking gut dysbiosis and NK cell exhaustion as promising biomarkers
Source: Front Immunol. 2025 Oct 31;16:1637098. doi: 10.3389/fimmu.2025.1637098 (PMC12615445; doi:10.3389/fimmu.2025.1637098)
Supplement: Supplementary file 1 [file Table1.docx]

***Supplementary 1. Calculation of exhaustion and dysbiosis scores***

The variables that were considered for the calculation of the NK cell exhaustion score, where the percentage of cells expressing the individual receptors PD-1, LAG-3, BTLA, TIM-3, TIGIT, and NKG2A, and their co-expression PD-1^+^BTLA^+^, PD-1^+^LAG-3^+^, PD-1^+^TIM-3^+^, PD-1^+^TIGIT^+^, TIGIT^+^TIM-3^+^, NKG2A^+^TIGIT^+^, PD-1^+^NKG2A^+^, and NKG2A^+^TIM-3^+^ on CD56^dim^ and CD56^bright^ NK cells.

For the dysbiosis score, the considered variables were α-diversity metrics (Shannon, Pielou, Simpson, and Strong indices) and the CLR-transformed relative abundances of bacterial taxa previously identified as expanded or depleted by ANCOM-BC analysis in CC patients in prior analyses (45 bacterial taxa).

Z-score calculation of each variable of microbiota and NK cell analysis:

$$Z_{x}=\frac{X-\overline{X_{HD}}}{{IQR}_{HD}}$$

Where:

$\boldsymbol{X}$ is the observed value in the variable of the sample.

$\overline{\boldsymbol{X}}$ is the median of the X-variable of the HD group.

$\boldsymbol{IQR}$ is the interquartile range of the variable X of the HD group, defined as:

$${IQR}_{HD}={P_{X}}_{75}{- P_{X}}_{25}$$

$P_{X}$ is the percentile of the X variable of the HD group.

For each NK receptor in CD56^dim^ and CD56^bright^ populations, a score was assigned based on the HD group percentiles:

$$ExS Z_{x} {CD56}^{dim}NK cell \left\{ \begin{aligned} If Z_{x}\leq{P_{z}}_{75},1 \\ If {P_{z}}_{75}<Z_{x}{\leq{P_{z}}_{90},2} \\ If Z_{x}>{P_{z}}_{90}, 3 \end{aligned} \right.$$

$${CD56}^{dim} NK cell ExS=\sum ExS Z_{x} {CD56}^{dim}NK cell\left\{ \begin{aligned} If ExS Z_{x}\leq P_{75exS},1 \\ If ExS P_{75exS}<Z_{x}{\leq P}_{90exS} \\ If ExS Z_{x}>P_{90exS},3 \end{aligned} \right.,2$$

$$ExS Z_{x} {CD56}^{bright} \left\{ \begin{aligned} If Z_{x}\leq{P_{z}}_{75},1 \\ If {P_{z}}_{75}<Z_{x}{\leq{P_{z}}_{90},2} \\ If Z_{x}>{P_{z}}_{90}, 3 \end{aligned} \right.$$

$${CD56}^{bright} NK cell ExS=\sum ExS Z_{x} {CD56}^{bright}NK cell\left\{ \begin{aligned} If ExS Z_{x}\leq P_{75ExS},1 \\ If ExS P_{75ExS}<Z_{x}{\leq P}_{90ExS} \\ If ExS Z_{x}>P_{90ExS},3 \end{aligned} \right.,2$$

$$Global NK cell ExS= \frac{ExS {CD56}^{dim} NK cell+ ExS {CD56}^{bright} NK cell}{2}$$

Where:

$\boldsymbol{P}_{\boldsymbol{z}}$ is the percentile of the $Z_{x}$variable of the HD group.

**ExS** is the exhaustion score.

$\boldsymbol{P}_{\boldsymbol{exS}}$ is the percentile of the variable **ExS** of the HD group.

Similarly, a dysbiosis score was assigned based on the percentiles of the HD group:

$$DyS Z_{x}\left\{ \begin{aligned} If {P_{z}}_{25}\leq Z_{x}\leq{P_{z}}_{75}, 1 \\ If {P_{z}}_{5}\leq Z_{x}<{P_{z}}_{25} or {P_{z}}_{75}<Z_{x}\leq{P_{z}}_{95},2 \\ If {Z_{x}<}{P_{z}}_{5} or Z_{x}>{P_{z}}_{95},3 \end{aligned} \right.$$

$DyS=\sum Ex Score Z_{x}\left\{ \begin{aligned} If {P_{DyS}}_{25}\leq Z_{x}\leq{P_{DyS}}_{75}, 1 \\ If {P_{DyS}}_{5}\leq Z_{x}<{P_{DyS}}_{25} or {P_{DyS}}_{75}<Z_{x}\leq{P_{Dys}}_{95},2 \\ If {Z_{x}<}{P_{DyS}}_{5} or Z_{x}>{P_{DyS}}_{95},3 \end{aligned} \right.$

Where:

$\boldsymbol{P}_{\boldsymbol{z}}$ is the percentile of the $Z_{x}$variable of the HD group.

**DyS** is the dysbiosis score.

$\boldsymbol{P}_{\boldsymbol{DyS}}$ is the percentile of the variable **DyS** of the HD group.
