# Supplementary material for: Microbiota–immune dysregulation in cervical cancer patients from Western Mexico: linking gut dysbiosis and NK cell exhaustion as promising biomarkers
Source: Front Immunol. 2025 Oct 31;16:1637098. doi: 10.3389/fimmu.2025.1637098 (PMC12615445; doi:10.3389/fimmu.2025.1637098)
Supplement: Supplementary file 2 [file Table2.docx]

| Statistical analysis of α-diversity | | | | |
| --- | --- | --- | --- | --- |
| Shannon^a^ | | | | |
| Group 1 | Group 2 | | *p* value | *q* value |
| HD | CC pre-tx | | 0.0385 | 0.0385 |
|  | CC post-tx | | <0.0001 | <0.0001 |
| CC pre-tx | CC post-tx | | 0.0053 | 0.0079 |
| Simpson^b^ | | | | |
| HD | CC pre-tx | | 0.0262 | 0.0262 |
|  | CC post-tx | | <0.0001 | <0.0001 |
| CC pre-tx | CC post-tx | | 0.0206 | 0.0206 |
| Pielou evenness^b^ | | | | |
| HD | CC pre-tx | | 0.0188 | 0.0188 |
|  | CC post-tx | | <0.0001 | <0.0001 |
| CC pre-tx | CC post-tx | | 0.0101 | 0.0152 |
| Simpson evenness^a^ | | | | |
| HD | CC pre-tx | 0.0039 | | 0.0039 |
|  | CC post-tx | <0.0001 | | <0.0001 |
| CC pre-tx | CC post-tx | 0.0018 | | 0.0026 |
| Strong^b^ | | | | |
| HD | CC pre-tx | 0.0131 | | 0.0172 |
|  | CC post-tx | <0.0001 | | <0.0001 |
| CC pre-tx | CC post-tx | 0.0172 | | 0.0172 |

**Supplementary Table 2A.** α-diversity in healthy donors (HD), patients before treatment (CC pre-tx), and patients after treatment (CC post-tx). Pairwise comparisons of α-diversity indices (Shannon, Simpson, Pielou evenness, Simpson evenness, Strong) were performed using ^a^ANOVA or ^b^Kruskal-Wallis followed by pairwise post-hoc tests with FDR correction. *P* and *q* values are shown.

**Supplementary Figure 2B.** Gut microbiota β-diversity in fecal samples from healthy donors (HD), CC patients before treatment (CC pre-tx), and CC patients after treatment (CC post-tx). Three-dimensional scatter plot obtained by PCoA using Jaccard, unweighted unifrac, and weighted unifrac, showing the distance between study groups in terms of β-diversity. Statistical analyses were performed using PERMANOVA to determine the statistical significance of the observed separations in the coordinate space. PCoA: Principal Coordinates Analysis, PERMANOVA: Permutational Multivariate Analysis of Variance.

| PERMANOVA values of β-diversity | | | | |  |
| --- | --- | --- | --- | --- | --- |
| Jaccard | | | | |  |
| Group 1 | Group 2 | Pseudo F | *p* value | *q* value |  |
| HD | | CC pre-tx | 1.298632 | 0.008 | 0.012 |
|  |  | CC post-tx | 2.423666 | 0.001 | 0.003 |
| CC pre-tx | CC post-tx | 1.30701 | 0.056 | 0.056 |  |
| Weighted unifrac | | | | |  |
| HD | CC pre-tx | 2.191282 | 0.032 | 0.048 |  |
|  | CC post-tx | 5.025288 | 0.002 | 0.006 |  |
| CC pre-tx | CC post-tx | 1.944779 | 0.059 | 0.059 |  |
| Unweighted unifrac | | | | |  |
| HD | CC pre-tx | 2.191282 | 0.032 | 0.048 |  |
|  | CC post-tx | 5.025288 | 0.002 | 0.006 |  |
| CC pre-tx | CC post-tx | 1.944779 | 0.059 | 0.059 |  |

**Supplementary Table 2C.** β-diversity in healthy donors (HD), cervical cancer patients before treatment (CC pre-tx), and after treatment (CC post-tx). Pairwise comparisons of community composition were assessed by PERMANOVA (Jaccard, Weighted UniFrac, Unweighted UniFrac) with FDR correction. Pseudo-F values, *p* values, and *q* values are shown.
