# Supplementary material for: Microbiota–immune dysregulation in cervical cancer patients from Western Mexico: linking gut dysbiosis and NK cell exhaustion as promising biomarkers
Source: Front Immunol. 2025 Oct 31;16:1637098. doi: 10.3389/fimmu.2025.1637098 (PMC12615445; doi:10.3389/fimmu.2025.1637098)
Supplement: Supplementary file 3 [file Table3.docx]

**Supplementary 3.** Calculation of microbiota ratios

The count tables obtained after taxonomic assignment were frequency filtered, as described in the Materials and Methods section. These count tables were then subjected to centered log-ratio transformation (CLR) and collapsed to the taxonomic level of phylum and species. Finally, the following equations were applied to transformed count tables for ratio calculations at the phylum level (equation 1) and at the genus level (equation 2). The calculations were performed for each sequenced sample.

$$\frac{Proteobacteria}{Firmicutes}=\frac{Proteobacteria}{\left( Proteobacteria+Firmicutes \right)}$$

$$\frac{Escherichia/Shigella}{Ruminococcus}=\frac{Escherichia/Shigella}{\left( Ruminococcus+Escherichia/Shigella \right)}$$

Therefore, ratios results can be interpreted in such a way that a value close to 1 indicates a greater relative abundance of the bacteria described in the numerator (*Proteobacteria* or *Escherichia*/*Shigella*), while a value close to zero shows a greater abundance of the bacteria indicated in the denominator (*Firmicutes* or *Ruminococcus*).
