# Supplementary figures and images for: Microbiota–immune dysregulation in cervical cancer patients from Western Mexico: linking gut dysbiosis and NK cell exhaustion as promising biomarkers
Source: Front Immunol. 2025 Oct 31;16:1637098. doi: 10.3389/fimmu.2025.1637098 (PMC12615445; doi:10.3389/fimmu.2025.1637098)

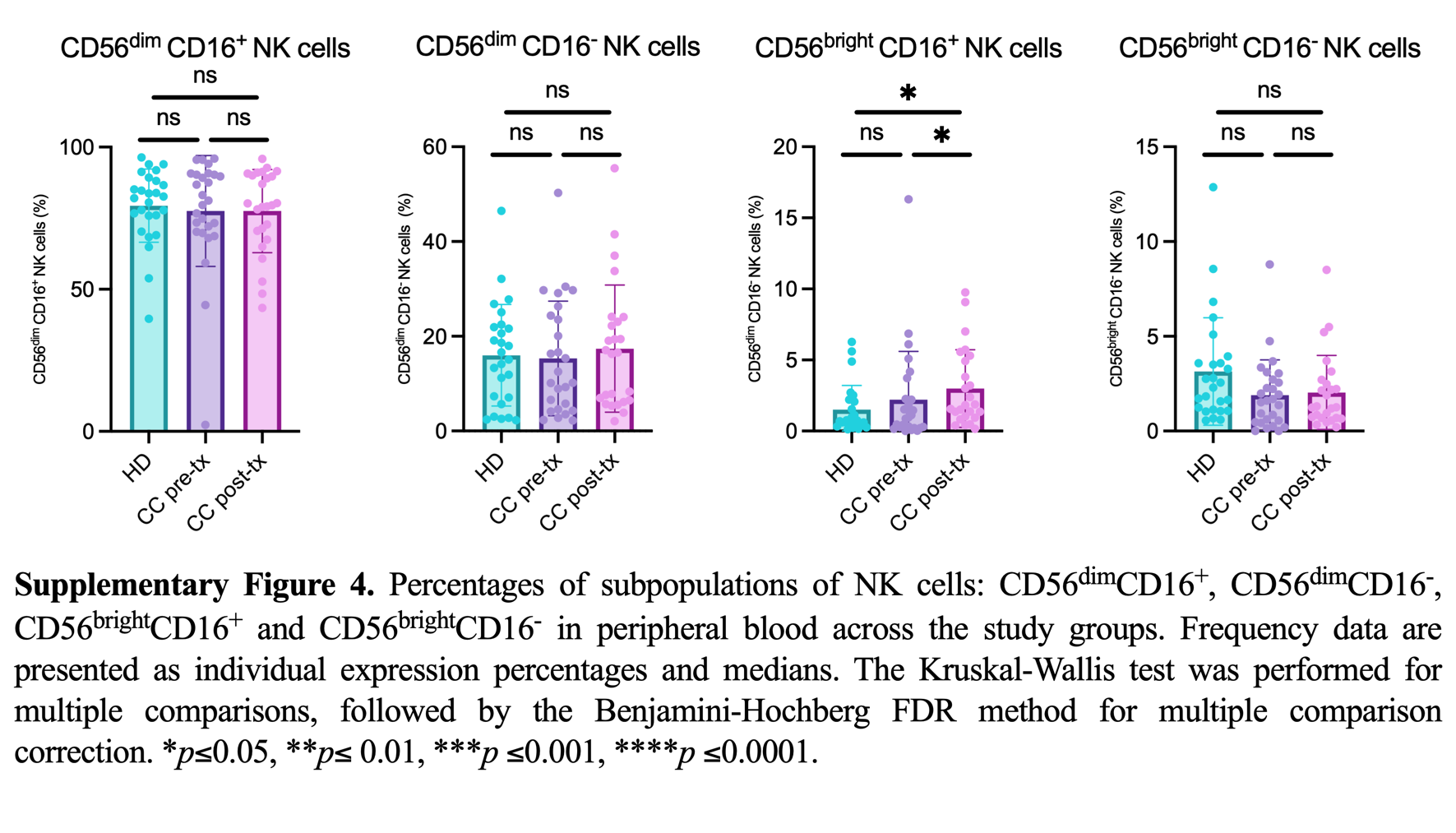

Supplement: Supplementary file 4 [file Image1.tiff]

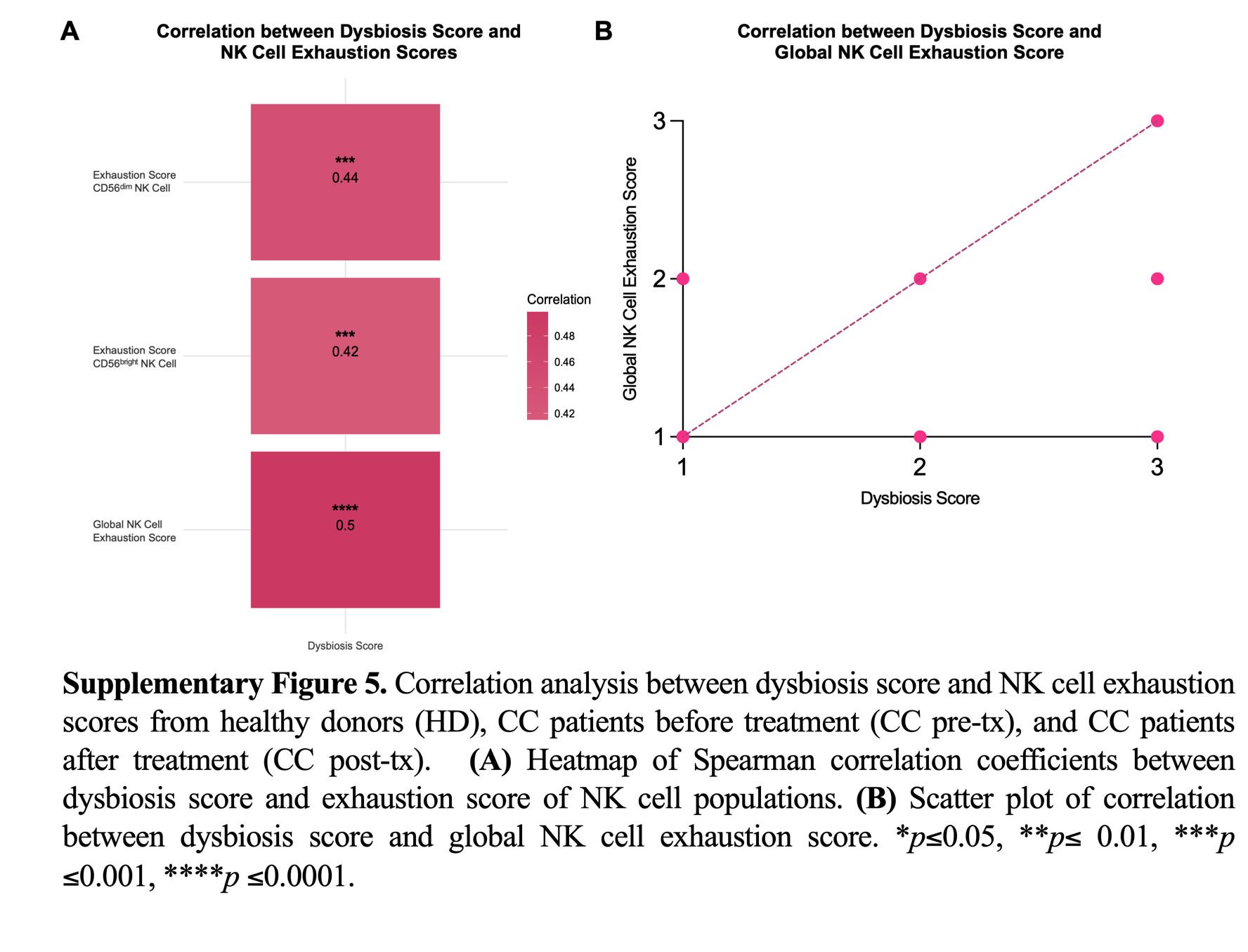

Supplement: Supplementary file 5 [file Image2.tiff]

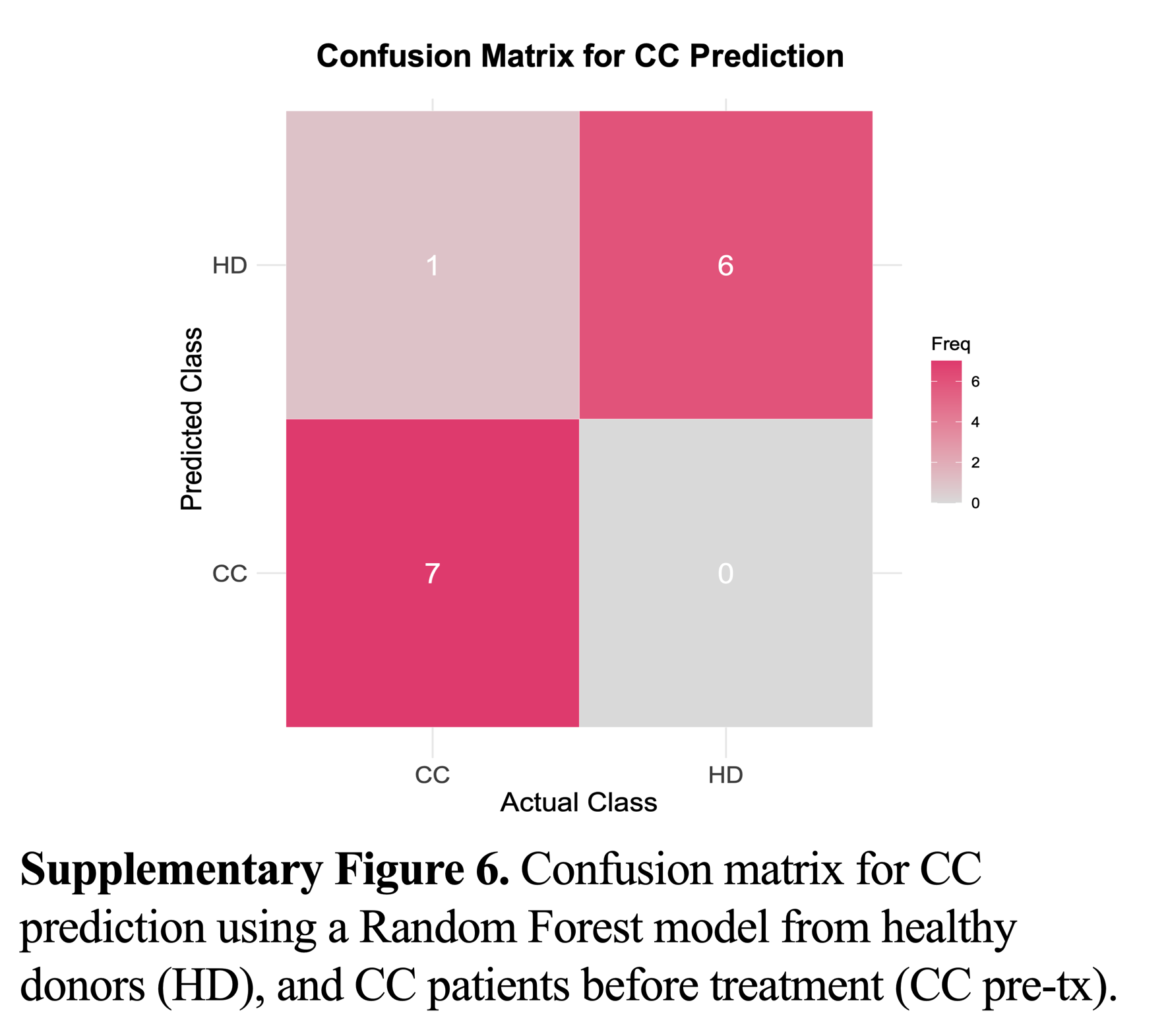

Supplement: Supplementary file 6 [file Image3.tiff]

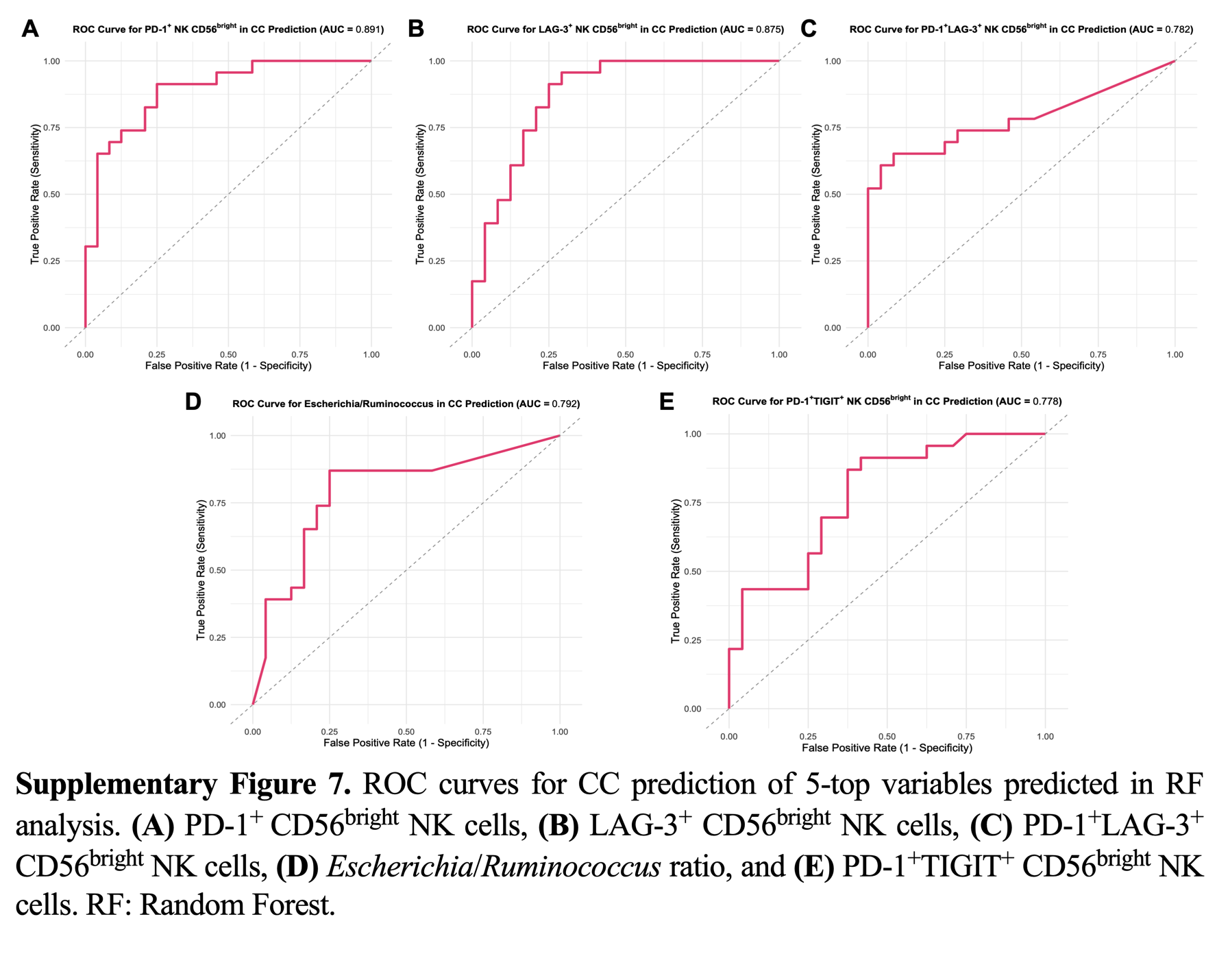

Supplement: Supplementary file 7 [file Image4.tiff]

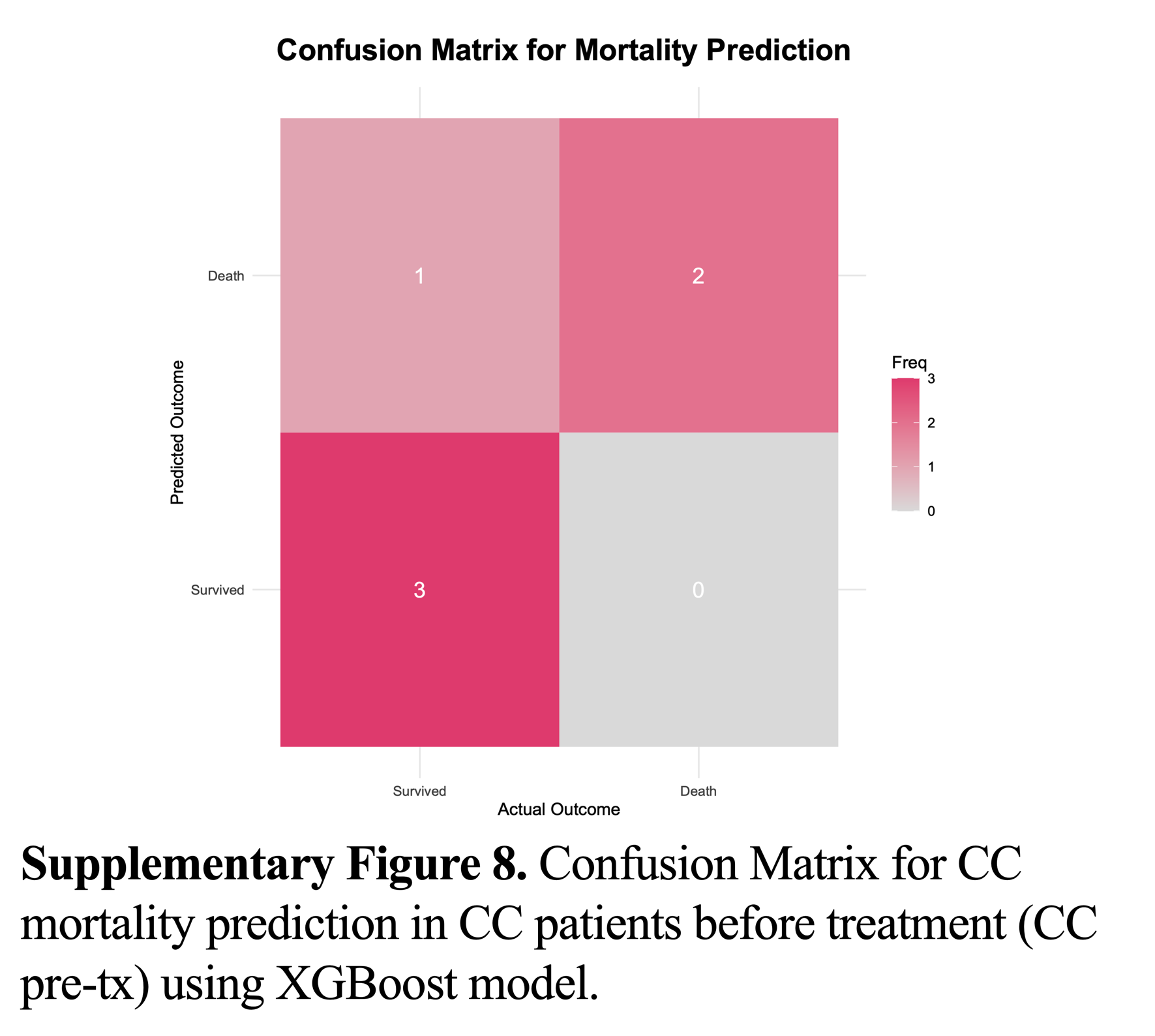

Supplement: Supplementary file 8 [file Image5.tiff]

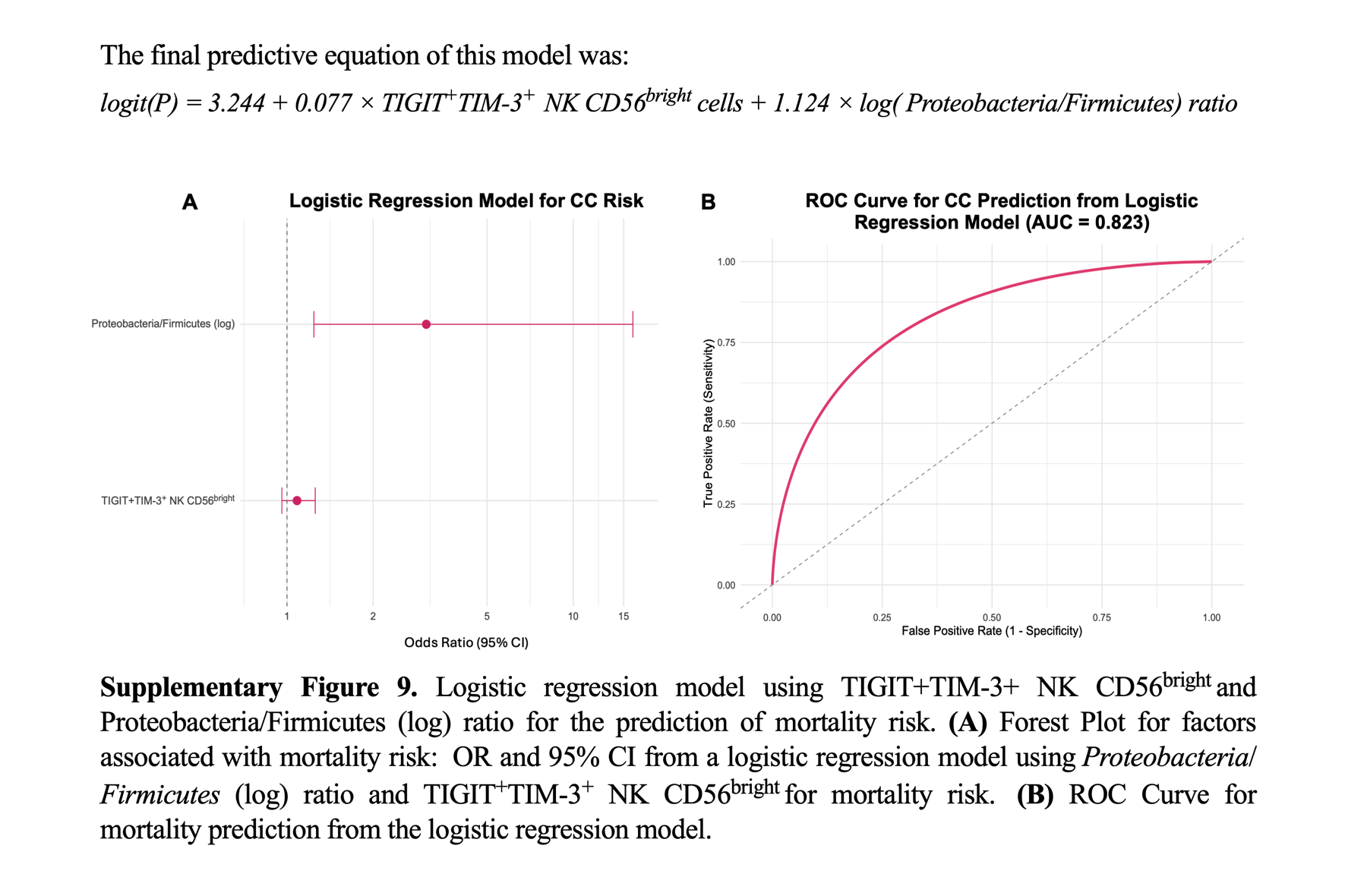

Supplement: Supplementary file 9 [file Image6.tiff]
